# Spatiotemporal dynamics of leptospirosis in Europe: a retrospective observational study with prospective projections

**DOI:** 10.1016/j.lanepe.2026.101671

**Published:** 2026-04-06

**Authors:** Angela Fanelli, Baptiste Alglave, Luca Caporaso, Alessandro Cescatti, Juan-Carlos Ciscar, Gregoire Dubois, Alessandro Dosio, Rosa M. Estevez-Reboredo, María Blazquez, Dolores Ibarreta, Andrea Mandrici, Emanuele Massaro, Rachel Lowe, Wojtek Szewczyk

**Affiliations:** a European Commission, Joint Research Centre (JRC), Seville, Spain; b Facultad de Veterinaria, Universidad Alfonso X el Sabio (UAX), Avenida de la Universidad 1, 28691, Villanueva de la Cañada, Madrid, Spain; c Université Bretagne Sud, Lab-STICC, 56000, Vannes, France; d European Commission, Joint Research Centre (JRC), Ispra, Italy; e National Research Council of Italy, Institute of BioEconomy, Rome, Italy; f Centro Nacional de Epidemiología, Instituto de Salud Carlos III (ISCIII), Madrid, Spain; g Consortium for Biomedical Research in Epidemiology and Public Health (CIBERESP), Madrid, Spain; h Escuela Nacional de Sanidad, Instituto de Salud Carlos III (ISCIII), Madrid, Spain; i Arcadia SIT S.r.l., 27029, Vigevano, Italy; j Barcelona Supercomputing Center (BSC), Barcelona, Spain; k Catalan Institution for Research and Advanced Studies (ICREA), Barcelona, Spain; l Centre on Climate Change and Planetary Health and Centre for Mathematical Modelling of Infectious Diseases, London School of Hygiene and Tropical Medicine, London, United Kingdom

**Keywords:** Leptospirosis, Climate change, Europe, Spatiotemporal modelling, Zoonotic disease, Epidemiological data

## Abstract

**Background:**

Leptospirosis is a widespread zoonotic disease caused by *Leptospira* spp., with a substantial burden on both human and animal health. Transmission occurs through contact with the urine of infected animals, with rodents serving as the main disease reservoir. Climate and environmental changes, including higher temperatures, heavy rainfall, urbanization, forest encroachment, and biodiversity loss are expected to alter rodent populations and disease dynamics, raising concerns about the increasing risk of leptospirosis in Europe. Despite this, no comprehensive risk map or modelling study has been conducted at the European scale. Given this, we aimed to quantify spatiotemporal patterns of leptospirosis risk in Europe and project future incidence rate under climate change scenarios.

**Methods:**

In this study, we present a Europe-wide spatiotemporal assessment of leptospirosis risk, integrating epidemiological, environmental and climate data. Using monthly leptospirosis cases from the European Surveillance System (TESSy) between 2010 and 2023, we developed a Bayesian hierarchical model to identify key risk factors, including the 3-month Standardised Precipitation Evapotranspiration Index (SPEI-3), mean temperature (Tmean), forest human nexus and mammal richness. Future changes in disease incidence rate were also investigated under two climate/socio-economic scenarios, namely the Shared Socioeconomic Pathways (SSP) 4.5 and 8.5.

**Findings:**

Over the 2010–2023 period, leptospirosis risk was higher in areas with elevated SPEI-3 (maximum relative risk [RR] = 1.62; 95% CrI: 1.20–2.10) and higher Tmean values (maximum RR = 5.28; 95% CrI: 2.65–11.12), both with a 1-month lag. Leptospirosis incidence rates were greater in warm coastal regions and densely populated areas. We observed a complex relationship between disease risk and the forest human nexus (maximum RR = 1.23; 95% CrI: 0.96–1.55), and a negative association with mammal species richness (minimum RR = 0.63; 95% CrI: 0.40–0.98), suggesting a potential dilution effect. Finally, under future climate scenarios, we found that leptospirosis incidence rate is projected to increase, particularly under high greenhouse gas (GHG) emissions, with larger rises expected in the long-term period (2081–2100) than in the short-term period (2041–2060).

**Interpretation:**

Climate and environmental factors can elevate the risk of leptospirosis with climate change projected to have a significant impact on future risk levels. Our findings underscore the need for proactive measures to mitigate the effects of climate-sensitive zoonoses, such as leptospirosis, across Europe. Integrating spatial and temporal disease models into early warning systems, alongside environmental management and climate mitigation strategies, can support timely interventions and enhance public health resilience.

**Funding:**

This research received no specific grant from any funding agency in the public, private, or not-for-profit sectors.

**Research in context**

**Evidence before this study**

Leptospirosis is a globally important but neglected zoonotic disease, primarily transmitted by rodents and influenced by climate and environmental conditions. We conducted a comprehensive search of Scopus and PubMed for English-language articles published from database inception to 07/02/2026, using the keywords (“leptospirosis” OR “leptospira”) AND (“rodent∗” OR “rat∗”) AND (“zoonosis” OR “zoonotic disease∗”) AND (“spatial model∗” OR “geospatial model∗” OR “risk mapping”) AND (“risk factor∗” OR “determinant∗” OR “predictor∗” OR “driver∗”). To date, most risk assessments and spatial modelling studies of leptospirosis have been limited to country-level analyses, predominantly in tropical regions where the disease burden is the highest. These studies have identified environmental, climate, socio-economic, and demographic factors as key drivers of disease risk. However, no comprehensive risk map or modelling study have been undertaken at the European scale, leaving substantial gaps in our understanding of the current geographic distribution of leptospirosis and the potential impact of future climate change on its dynamics.

**Added value of this study**

We present the first Europe-wide spatiotemporal modelling of leptospirosis risk, integrating epidemiological data with environmental and climate predictors. By employing a Bayesian hierarchical framework, we identified the key drivers shaping leptospirosis risk across Europe and projected how climate change may influence its future distribution. Our findings reveal positive associations between higher 3-month Standardised Precipitation Evapotranspiration Index (SPEI-3) and mean temperature (Tmean) values, both lagged by one month, and increased leptospirosis risk. We also observed a negative relationship between mammal richness and leptospirosis incidence rates, suggesting a potential dilution effect, whereby higher biodiversity may reduce transmission opportunities, and a complex relationship with forest human nexus. Projections under future climate scenarios, Shared Socioeconomic Pathways (SSP) 4.5 and 8.5, indicate a statistically significant increase in leptospirosis incidence rates, with greater magnitude under high-emission scenarios. By applying a multidisciplinary approach that integrates diverse epidemiological, environmental, and climate datasets, this study offers critical evidence to inform surveillance strategies, guide public health preparedness, and support targeted interventions addressing climate-sensitive zoonoses in Europe.

**Implications of all the available evidence**

Our findings, together with existing evidence, underscore the urgent need for proactive measures to mitigate the impacts of climate-sensitive zoonoses in Europe. Incorporating spatiotemporal disease models into early warning systems could help anticipate outbreaks and enable timely interventions. In the longer term, effective environmental management and climate mitigation strategies aimed at lowering greenhouse gas (GHG) emissions can help address the underlying environmental and climate drivers of leptospirosis, thereby strengthening overall public health resilience. These insights can guide policy decisions and public health strategies, supporting better preparedness and response to the evolving threat of leptospirosis under climate change, and enabling the design and implementation of effective measures to reduce infections. Nonetheless, the integration of animal health data remains a key area for improvement, constrained by the current lack of harmonized information on leptospirosis occurrence in animal reservoirs.

## Introduction

Leptospirosis is a neglected zoonotic disease caused by *Leptospira* spp., with major impacts on human and animal health across the globe.^1^ A systematic review published in 2015 estimated that there could be one million leptospirosis cases and 60,000 related deaths per year globally.^2^ Transmission occurs through direct or indirect contact with the urine of infected animals, most commonly rodents, which represent the main reservoir. Domestic animals, livestock, and wildlife species also play an important role.^1^ Despite its importance, leptospirosis remains underdiagnosed and underreported, which reinforces its status as a neglected disease.^1^^,^^2^

The disease is prevalent in tropical and subtropical regions, where environmental and socio-economic conditions favour transmission, and it is recognised as one of the world's most widespread emerging zoonoses.^2^ In contrast, the epidemiological profile of leptospirosis in Europe differs substantially,^2^^,^^3^ with human infections frequently associated with occupational or recreational exposure to contaminated soil or water.^1^ Although the overall incidence is lower than in tropical regions, cases are more frequently linked to specific high-risk groups and activities. Occupational exposure, including farmers, sewer workers, veterinarians, and water–recreational activities represent the primary routes of infection.^3^ These exposure patterns highlight that, unlike in tropical and subtropical settings where infection can be widespread across communities, leptospirosis in Europe tends to affect individuals with particular behavioural or occupational risk factors.

The true burden of leptospirosis is likely underestimated due to diagnostic challenges and reporting limitations.^2^ As a rodent-borne, climate-sensitive infection, leptospirosis is strongly influenced by climate drivers such as rainfall, flooding, soil moisture, and temperature, all of which are projected to change under future climate scenarios.^4^^,^^5^ Climate change is already reshaping rodent populations and the dynamics of rodent-borne diseases,^4^^,^^6^ emphasising the need to understand how leptospirosis risk may evolve in Europe in the future. Recent flooding events, such as the severe 2024 DANA storm in Valencia, where DANA (*Depresión Aislada en Niveles Altos*) refers to a Mediterranean upper-level cold low that can generate intense and sudden heavy rainfall, along with other extreme weather episodes, have heightened concerns about the potential re-emergence of the disease under changing climate conditions. Moreover, change in land use across Europe, such as the recent increase in unused or abandoned land and the decline in agricultural and forested areas,^7^ are likely to alter wildlife habitats and human exposure patterns, influencing the environmental circulation of *Leptospira* spp.

To date, risk assessments and spatial models of leptospirosis have been largely limited to country-level studies,^8^ often conducted in tropical regions where the burden is highest.^9^, ^10^, ^11^ These studies identified environmental, climate, socioeconomic, and demographic factors as drivers of the disease.^8^ However, no comprehensive risk map or modelling study has been undertaken at the European scale, leaving substantial gaps in our understanding of the current geographic distribution of leptospirosis, its reservoir species, and the potential impact of climate change on its dynamics.

Until 2005, leptospirosis in animals was classified as a List B disease by the World Organization for Animal Health (WOAH, formerly OIE),^12^ which required testing of animals involved in trade. Today, leptospirosis is no longer on the WOAH list, limiting comprehensive surveillance and preventing a full One Health approach due to the lack of systematic data on animal infections. Human leptospirosis data remain available through the European Surveillance System (TESSy) of the European Centre for Disease Prevention and Control (ECDC), supporting disease surveillance and preparedness in Europe.

This work presents the first Europe-wide spatiotemporal modelling of leptospirosis risk, integrating epidemiological human data with environmental and climate predictors. We identified the key drivers shaping leptospirosis risk across the continent, characterised the disease spatial and temporal patterns, and projected how climate change may influence its future distribution. By producing the first continental-scale risk map, this work provides essential insights for surveillance, public health preparedness, and it may help the design of targeted interventions to mitigate the impacts of climate-sensitive zoonoses in Europe.

## Methods

### Data

In this study, we analysed monthly leptospirosis cases between January 2010 and December 2023 officially reported by Member States to the ECDC and stored in TESSy. Cases were aggregated at the NUTS 3 level. NUTS, the Nomenclature of Territorial Units for Statistics, is the European Union's standardised regional classification system that divides the economic territory into three hierarchical levels (NUTS 1–3), ranging from larger to smaller regions. The analysis was restricted to 1499 NUTS 3 regions across 37 countries with complete covariate data. Annual population estimates were derived from the Global Human Settlement Layer (GHSL) to calculate disease incidence rates.

Four potential climatic and environmental drivers were considered. Meteorological data included the monthly 3-month Standardised Precipitation–Evapotranspiration Index (SPEI-3) and monthly 2-m air temperature (Tmean). SPEI-3 was derived from the SPEIbase, developed by the Climatology and Climate Services Laboratory. The SPEI provides a standardised measure of drought intensity, where positive and negative values denote wet and dry anomalies, respectively. Tmean was retrieved from ERA5-Land, a climate reanalysis dataset produced by the European Centre for Medium-Range Weather Forecasts (ECMWF). It is important to note that the SPEIbase is based on the daily data from ERA5, as detailed in the corresponding dataset publication.^13^

Data on the forest human nexus were obtained from Massaro et al.,^14^ available at 5-year intervals and interpolated to annual resolution. The forest human nexus is a spatial indicator that integrates forest area per capita, forest accessibility, and population density to provide a geospatial assessment of human–forest relationships, serving as a proxy indicator for exposure to wild areas and potential human–wildlife interactions.^14^ Its values range from 0 to 1, where 1 reflects the highest possible human forest proximity (e.g., one person living in an area entirely surrounded by forest), and 0 indicates a landscape with neither forest nor human presence.^14^ Mammal species richness data are available from The International Union for Conservation of Nature's (IUCN) Red List Spatial Dataset, and were processed following the approach developed by the Knowledge Center for Biodiversity-Global Biodiversity Data (KCBD-GBD; ex-DOPA) unit of the JRC.^15^ Mammal richness is a count of the number of species occurring in an area.

For future climate conditions, we used projections from the NEX-GDDP dataset hosted by NASA's Socioeconomic Data and Applications Center (SEDAC) for the SPEI-3, and from the NASA Center for Climate Simulation (NCCS) for Tmean. The NASA-GDDP dataset provides climate projections derived from CMIP6 global models that have been bias-adjusted to correct systematic errors and statistically downscaled to a finer spatial resolution, making them suitable for regional analysis. All covariates were spatially averaged at the NUTS 3 level to ensure alignment with the notified case data.

### Model development

We fitted a spatiotemporal Bayesian hierarchical model to monthly notified leptospirosis cases across the 1499 NUTS 3 regions. Case counts followed a negative binomial distribution to address overdispersion, with the log of the population included as an offset. Model inference was conducted using the Integrated Nested Laplace Approximations (INLA) in R version 4.5.0.^16^ The baseline model included monthly autocorrelated random effects to capture seasonality, modelled as a first-order random walk (rw1); year-level random effects to account for inter-annual variability, modelled as independent and identically distributed Gaussian effects (iid); and NUTS 3–level spatial random effects to address spatial autocorrelation, modelled using the modified Besag–York–Mollié (BYM2) specification. Penalized Complexity (PC) priors were applied as weakly informative priors, with parameters set to α = 0.01 and *u* = 1. We fitted the above random effects-only model as a baseline and conducted model selection to develop a model including the covariates as smooth terms, modelled as a second-order random walk (rw2). Pairwise correlations between covariates were low (|r| < 0.3) (see Supplementary Material). Both SPEI-3 and Tmean were lagged by one month to describe the delayed dependencies between disease incidence rates and climate factors. The choice of a 1-month lag was based on exploratory analyses which indicated that lag-1 terms were consistently the most strongly associated with leptospirosis incidence for both SPEI-3 and Tmean. Lag selection was informed by a preliminary exploratory analysis using distributed lag non-linear models (DLNMs) to assess potential non-linear and delayed associations between leptospirosis incidence rate, Tmean, and SPEI across lags 0–6 months. For the SPEI, we explored 0.5-, 1-, 3-, and 6-month intervals before selecting SPEI-3. Both single-lag and cumulative effects over multiple months were examined, revealing a peak in the relative risk of leptospirosis at a 1-month lag, justifying the use of 1 month lagged covariates in further model selection. To assess the extent to which the covariates account for seasonality and interannual variability in leptospirosis dynamics, we conducted a sensitivity analysis by removing each covariate of interest from the full model and comparing the monthly and yearly random effects from the candidate model and the baseline model.

The final model was chosen by systematically comparing models of increasing complexity (in terms of both input covariates and model structure) against the baseline. Model performance was evaluated using standard goodness-of-fit and leave one-out validation metrics, including the Deviance Information Criterion (DIC) and the mean cross-validated log score. The DIC assesses model accuracy while penalizing excessive complexity through the number of effective parameters, whereas the log score evaluates predictive performance by iteratively excluding one data point at a time. For both metrics, lower values indicate superior model fit. Following recent advances in validation of INLA models,^17^ we also applied a leave-one-observation-out cross-validation (LOOCV) and leave-group-out cross-validation (LGOCV), the latter with the number of levels set to 3. The resulting log-scores were averaged to provide a single summary statistic of predictive capability. Exponentiating this value maps it back into the probability scale, offering a more intuitive interpretation: it represents the average probability the model assigns the true observed values to the data points. It is worth noting that in temporally structured models, the LOOCV technique primarily evaluates interpolation performance (i.e. how well the model reconstructs missing values within the observed time series). By contrast, LGOCV assesses the model capability to forecast the response variable at future time points.^17^

### Spatiotemporal patterns and key drivers of leptospirosis

The best-fitting model was used to assess the relationship between the covariates and leptospirosis incidence rate, and to predict monthly incidence per 100,000 people over the entire study period (168 months) with 95% credible intervals (CrI). As measures of uncertainty, we calculated the standard deviation (SD) and coefficient of variation (CV) of the average monthly estimates. We also developed monthly risk maps on a probability scale, derived from the classical epidemiological risk equation of the form $R_{t}=1-e^{\left(-I_{t}\right)}$ where the risk $R_{t}$ is defined as the proportion of the at-risk population affected during a period *t* ∈ [t0, t'], based on the incidence rate $I_{t}$. Finally, we reclassified the risk level into five categories: very low [0, 0.15), low [0.15, 0.3), medium [0.3, 0.45), high [0.45, 0.6), and very high [0.6, 1].

### Projected impacts of climate change on leptospirosis

To assess the potential influence of climate change on leptospirosis, we compared monthly incidence rates between a historical-reference period and future projections based on SSP2-4.5 and SSP5-8.5 scenarios. For future climate conditions, we used projections from five selected NEX-GDDP (see Supplementary Material for model section). SSP2-4.5 represents an intermediate greenhouse gas (GHG) emissions pathway, with CO_2_ emissions remaining near current levels until mid-century, then gradually declining but not reaching net zero by 2100. This scenario corresponds to a radiative forcing of 4.5 W/m^2^, with a projected warming of approximately 2.0 °C by 2041–2060 and 2.7 °C by 2081–2100.^18^ SSP5-8.5 represents a very high GHG emissions pathway, characterized by a tripling of CO_2_ emissions by 2075, sustained fossil fuel dependence, and rapid economic expansion. It corresponds to a radiative forcing of 8.5 W/m^2^, with projected warming of about 2.4 °C by 2041–2060 and 4.4 °C by 2081–2100.^18^ It is important to note that SSP scenarios account not only for CO_2_ emissions, which is the most important GHG, but also for broader socio-economic changes, such as population growth, gross domestic product (GDP), and land use change, and that emissions are represented as cumulative totals (not per-capita).^19^

Before running the future scenarios, we validated the model results by comparing results using first the observed climate data from SPEIbase v.2.10 and ERA5, and second the climate data from the bias-adjusted NEXGDPP models for the reference period (2010–2023). Results showed that the disease incidence was very similar between these simulations (see Supplementary Materials). Because our reference period was relatively short (2010–2023, 168 monthly time steps), we extended it for the 72 months preceding January 2010, resulting in a continuous time series starting in January 2004. This historical period (2004–2009) included the observed climate data, namely SPEI-3 from SPEIbase v.2.10 and Tmean from ERA5. The extension provided a 20-year baseline (240 months), comparable with the short-term future period (January 2041–December 2060) and long-term future period (January 2081–December 2100). Using a 20-year window is common practice in climate studies to adequately capture natural variability.

For this analysis, we applied a modelling framework that isolates the effects of climate while controlling for other covariates through offsets. While the climate variables were allowed to vary on a monthly timescale, the remaining variables were kept fixed. Population was fixed at its mean per NUTS 3 over 2010–2023, and regional heterogeneity was captured by averaging the random effects of environmental covariates across the study period. The disease incidence was computed for the extended time series (2004–2023, 2041–2060 and 2081–2100), then averaged monthly by model, scenario, NUTS 3, and period. Results were further averaged across the five NEX-GDDP models, and SD and CV were computed as measures of uncertainty. To summarise projected changes relative to the historical-reference period, we fitted linear models to compare future projections of disease incidence with those of the historical-reference baseline. This approach quantifies whether the model predicts a consistent increase or decrease in incidence across months and regions. Percentage changes in estimated incidence rates between future and historical-reference periods were also calculated for interpretability.

### Healthcare system vulnerability

We conducted a simple assessment of vulnerability to leptospirosis, considering the health system capacity of each country, proxied by the annual number of hospital beds per 100,000 inhabitants from EUROSTAT. For each country and year of the reference period (2010–2023), we calculated the average monthly incidence rate and compared it with the annual number of hospital beds per 100,000 inhabitants using a linear model to test for significant association. This analysis serves as an indicator of healthcare capacity, helping to contextualize how differences in healthcare resources may influence the ability to respond to leptospirosis outbreaks, rather than suggesting a direct causal link between bed availability and incidence.

### Ethics statement

Ethical approval was not required for this study because only anonymized data was used. Consent was waived as no identifiable personal information was used.

### Role of the funding source

The authors received no financial support for the research and authorship of this article.

## Results

The dataset included 3930 cases reported between 2010 and 2023, with the peak season occurring between August and September (see Supplementary Material). The average number of cases per year was 281, while the average number of NUTS 3 regions reporting cases per year was 140. The total cases and the number of region reporting cases per year showed a notable increasing trend over time (see Supplementary Material).

### Model development

The best-fitting model, which outperformed all other combinations by reducing the DIC, was the one that included all covariates [SPEI-3, Tmean, forest human nexus, mammal richness] (see Supplementary Material). On average the probability that the model assigns the correct outcome when predicting unseen data points in the LOOCV and LGOCV (e.g., correctly predicting the exact observed disease incidence rate) was for both measures 64%, indicating a high predictive capability given the strict criterion of exact matching (i.e. a result is counted as correct only when the model reproduces the exact observed incidence rate, with no tolerance threshold or partial-match scoring). Unlike commonly used accuracy metrics such as the area under the curve (AUC) or dependent-threshold metrics like sensitivity, specificity, and correct classification rate, typically applied to binomial (0–1) outcomes, these metrics assess the model's ability to exactly reproduce the observed incidence rate. This represents a far more stringent criterion than threshold-based classification. Under this definition, an accuracy of 64% is relatively high, as predicting exact incidence values is substantially more demanding than predicting categories or binary outcomes.

To assess the contribution of climate covariates to seasonality, we analyzed how the exclusion of Tmean and SPEI-3 affected the monthly random effects. When Tmean was excluded, the seasonal term absorbed much of the variation that would otherwise be explained by Tmean. In contrast, adding SPEI-3 only marginally reduced the contribution of the monthly random effects (see Supplementary Material). Regarding the inter-annual variability, in some years (e.g., 2010, 2014, 2022) the inclusion of SPEI-3 in the model decreased the contribution of the yearly random effects in accounting for inter-annual variability (see Supplementary Material).

### Spatiotemporal patterns and key drivers of leptospirosis

**Fig. 1:**
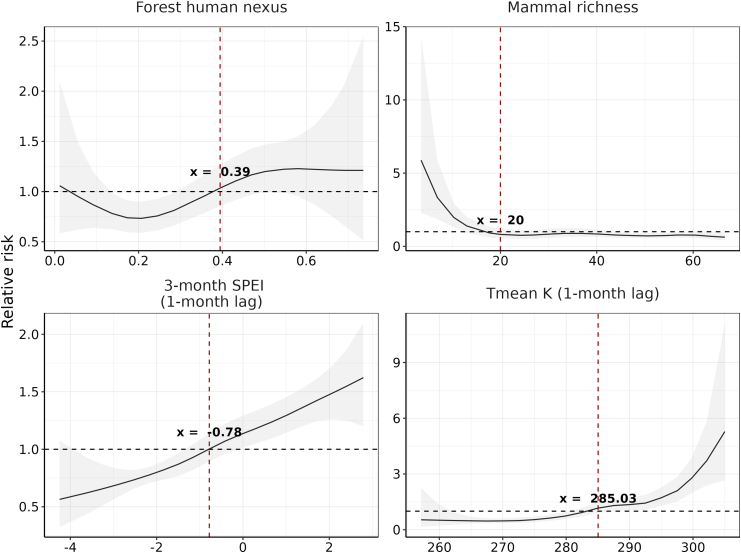
**Posterior marginal nonlinear marginal effects (specified as second-order random walks) from the best-fitted model shown on the relative risk scale, with lines and ribbons showing posterior mean and 95% CrI**.

The analysis of the best-fitting model revealed associations between climate factors and leptospirosis incidence rates. Specifically, we found that higher SPEI-3 values lagged by one month (>−0.78) and Tmean lagged by 1 month (>285 K, equivalent to 11.85 °C) were associated with an increased risk of leptospirosis. The relationship between the forest human nexus and leptospirosis incidence rates is complex and characterized by high uncertainty, as reflected by the wide 95% CrI. While our results indicate that leptospirosis risk increases as humans are in closer proximity to forests with a greater association observed when the forest human nexus exceeds 0.39, the relationship is nonlinear and levels off at higher values. Conversely, we found a negative relationship between mammal richness and leptospirosis incidence rate, which becomes significant at values < 20. The maximum and minimum relative risk [RR] and associated values for every covariate are shown in Table 1 and Fig. 1.

**Table 1:** Minimum and maximum model-estimated relative risks (RRs) and 95% credible intervals (CrIs) of leptospirosis by climate and environmental covariates.

Covariate	Risk type	Covariate value	RR	95% CrI
Forest human nexus	Minimum	0.21	0.73	0.59–0.90
	Maximum	0.58	1.23	0.96–1.55
Mammal richness	Minimum	66.56	0.63	0.40–0.98
	Maximum	3.55	5.88	2.30–14.32
SPEI-3 (1-month lag)	Minimum	−4.25	0.56	0.32–1.07
	Maximum	2.79	1.62	1.20–2.10
Tmean K (1-month lag)	Minimum	267.0	0.47	0.36–0.62
	Maximum	305.0	5.28	2.65–11.12

RR corresponds to the minimum and maximum values of the model-estimated risk surface for each covariate, along with the covariate value at which these extrema occur.

**Fig. 2:**
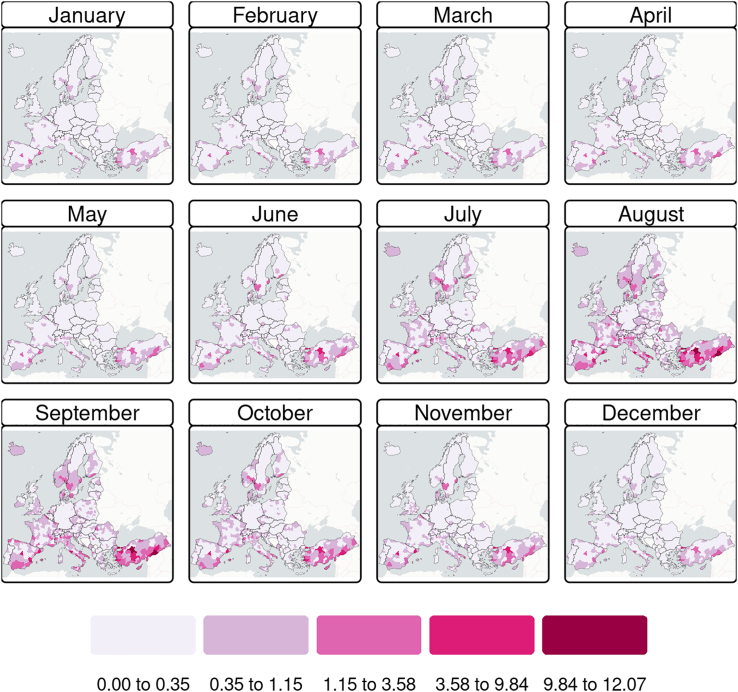
**Spatial distribution of leptospirosis incidence rates (cases per 100,000 population) across Europe at the NUTS 3 level, based on monthly averages for the reference period 2010–2023**.

Our results suggest that higher leptospirosis incidence rates are likely to occur in highly populated and warm regions, particularly in coastal zones (Fig. 2). The uncertainty is generally low but peaks regionally and in winter, suggesting local factors may strongly affect the incidence precision (see Supplementary Material).

**Fig. 3:**
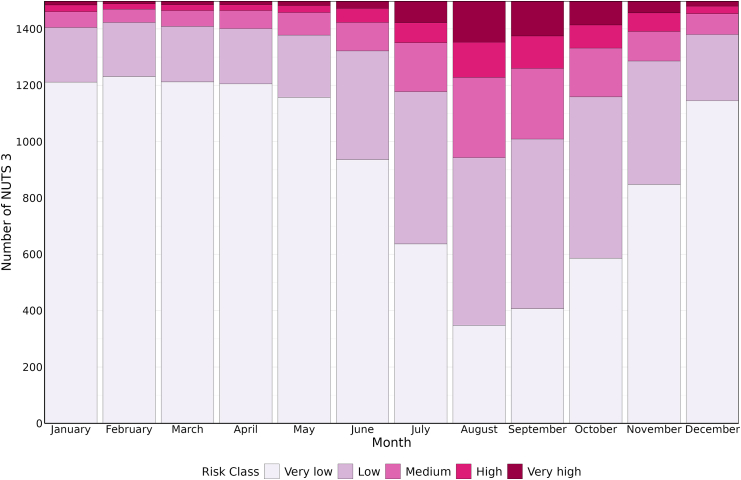
**Monthly number of NUTS 3 by leptospirosis risk category (total number of NUTS** = **1499, reference period 2010**–**2023)**.

Fig. 3 shows how the number of NUTS 3 in different risk categories changes by month, with the number of NUTS 3 at high and very high risk increasing towards the end of spring and throughout summer, peaking in August as the month with the highest number of NUTS classified as very high risk. Additional details are presented in the Supplementary Material.

### Projected impacts of climate change on leptospirosis

**Fig. 4:**
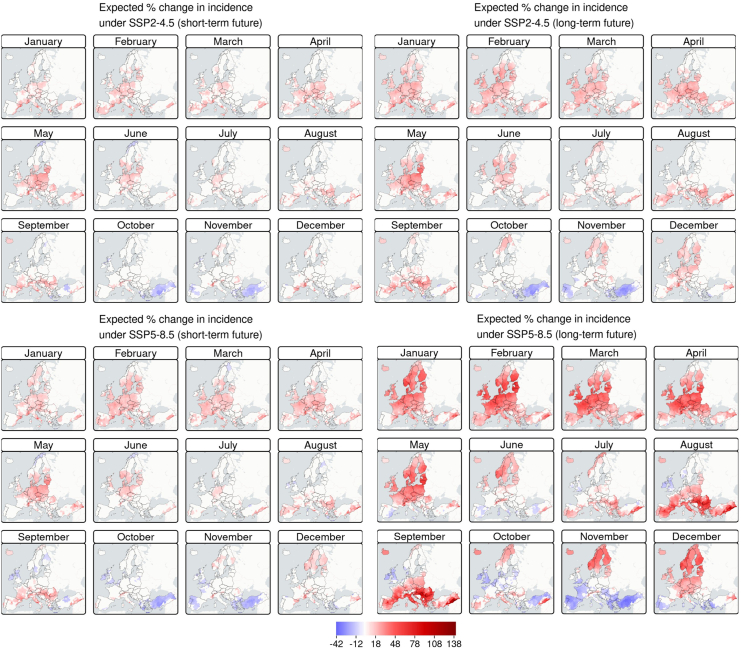
**Significant projected percentage changes in leptospirosis incidence rate under SSP2-4.5 and SSP5-8.5 for both short-term (2041**–**2060) and long-term (2081**–**2100) futures.** Only statistically significance differences (p-value<0.05) are shown, with non-significant differences shaded white.

To investigate how climate change may influence the burden of leptospirosis, we assessed shifts in monthly climate-driven incidence rate between a historical-reference period (2004–2023) and two future horizons: the short term (2041–2060) and the long term (2081–2100). Analyses were conducted under SSP2-4.5 and SSP5-8.5 using five NEX-GDPP models selected to capture high climate variability (see Supplementary Material for details of model selection). Across all NEX-GDDP models, the average uncertainty remains generally low, with lower values under SSP2-4.5. Conversely, projections under SSP5-8.5 exhibit greater variability, especially over the long term (see Supplementary Material). This suggests that high GHG emission futures are associated not only with higher incidence rates but also with greater projection uncertainty, likely reflecting the wider range of climate covariates in these scenarios, which are insufficiently represented in the historical-reference data. Across all NUTS 3–by–month combinations (1499 × 12 = 17,988), the estimated incidence rate was generally higher in the long-term future than in the short term under the same SSP scenario: 72% (71–73 95% confidence interval [CI]) of NUTS 3-month combinations under SSP2-4.5 and 71% (70–71 95% CI) under SSP5-8.5. When comparing SSP for the same period, incidence under SSP5-8.5 exceeded that of SSP2-4.5 in 58% (57–59 95% CI) of NUTS 3-month combinations for the short-term future and in 67% (66–68 95% CI) for the long-term future. Regression analyses, considering the historical-reference period as a baseline, confirmed these patterns. The proportion of NUTS 3 regions with a statistically significant positive increase in the disease incidence rate was consistently greater under SSP5-8.5 than under SSP2-4.5, with stronger effects observed in the long-term future in both scenarios. It is worth noting that the projections reveal a heterogeneous pattern of changes in disease incidence rates, with both increases and decreases varying across NUTS 3, months, and scenarios. In fact, there were also instances where a significant decrease in the disease incidence rate was detected; these cases represented only a small proportion of NUTS 3 by month combinations. These findings are shown in Fig. 4.

**Fig. 5:**
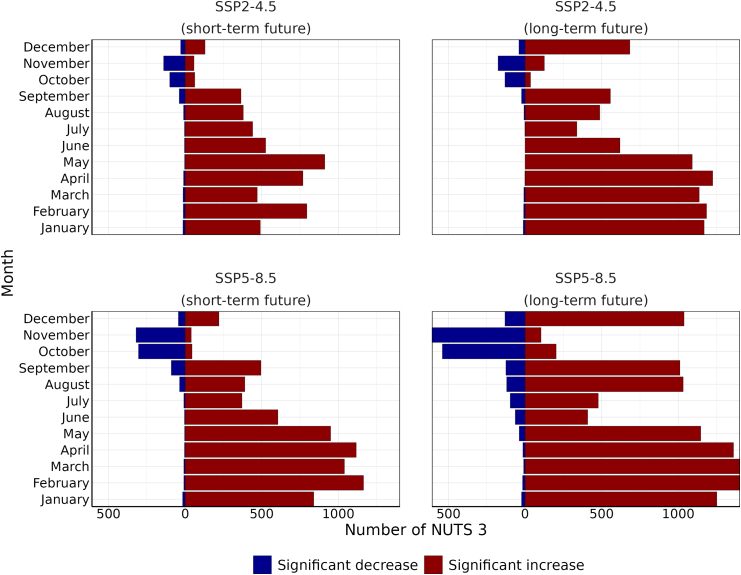
**Number of NUTS 3 regions with statistically significant changes in leptospirosis incidence rates under SSP2-4.5 and SSP5-8.5 for both short-term (2041–2060) and long-term (2081–2100) futures**.

Across both SSPs and future periods, significant increases were most pronounced during winter and spring (January to May), often affecting more than 70% of NUTS 3 regions (Fig. 5). The peak months varied by scenario and horizon: for SSP2-4.5, May showed the highest proportion in the short-term future (61%, 58–63 95% CI) and April in the long-term future (81%, 80–84 95% CI); for SSP5-8.5, February was highest in the short-term future (78%, 76–80 95% CI), while both February (94%, 92–95 95% CI) and March (94%, 92–95 95% CI) dominated in the long-term future (Fig. 5). During summer (June–August), the number of NUTS 3 with significant increases gradually declines, while the number of regions experiencing significant decreases remains generally low (Fig. 5). In autumn (October–November), a transition occurs: the number of NUTS 3 regions with statistically significant increases declines, while those with statistically significant decreases rise (Fig. 5). This pattern is especially pronounced under the SSP5-8.5 long-term scenario, where 36% (34–38 95% CI) and 40% (38–43 95% CI) of NUTS 3 regions are affected by a significant decrease in October and November respectively (Fig. 5). This pattern may indicate a possible seasonal shift in disease dynamics. However, it is important to note that these results represent statistically significant monthly increases or decreases relative to the historical-reference baseline (2004–2010), rather than the projected incidence rate of the disease itself. When examining the estimated incidence rate (cases per 100,000), the seasonality of the disease is characterised by the highest incidence rate in August and September, consistent with the reference period (see Supplementary Material).

### Healthcare system vulnerability

We conducted a basic assessment of leptospirosis vulnerability, taking into account each country's health system capacity, proxied by the number of hospital beds per 100,000 population. Preliminary analysis using a linear model revealed a significant negative association between hospital bed availability and the mean monthly incidence rate of leptospirosis ($\beta_{1}$ = −0.312, SE = ± 0.07, p-value <0.001), suggesting that countries with more limited healthcare infrastructure may be at greater risk of the disease.

## Discussion

We employed a spatiotemporal modelling approach to investigate the drivers of monthly leptospirosis incidence rates and to project changes in risk across Europe considering future climate scenarios. To our knowledge, this is the first study to examine the disease at a continental scale. Previous research has largely been confined to country-level analyses, predominantly in tropical areas.^9^, ^10^, ^11^ Although leptospirosis is generally regarded as a disease of low burden in Europe,^2^^,^^3^ understanding how climate and environmental factors shape its distribution and could drive significant changes in the disease incidence rate is crucial, particularly in the context of global warming.

Spatiotemporal epidemiological approaches hold considerable promise for improving our understanding of leptospirosis dynamics and for guiding effective interventions. However, their validity is highly dependent on data quality.^8^ As highlighted in a recent review of spatial epidemiological methods for leptospirosis, most studies rely on notification data derived from passive surveillance systems, which are likely to underestimate the true incidence. Nevertheless, such data remain more feasible to collect than large-scale eco-epidemiological surveys.^8^ For a disease prone to underreporting, like leptospirosis, this represents a notable challenge.

In our study, we addressed these limitations by applying a Bayesian geostatistical framework that accounts for both spatial and temporal autocorrelation and provides explicit estimates of uncertainty. However, underreporting is still likely to influence the spatial patterns estimated. Variation in diagnostic capacity, clinician awareness, and reporting practices across Europe means some regions may appear “low risk” due to limited case detection. While the BYM2 model helps accommodate such heterogeneity, it cannot fully account for systematic surveillance gaps, which may also bias environmental associations where surveillance quality aligns with climate or socioeconomic gradients. Future models could be further refined by incorporating behavioural and socioeconomic variables.

Additionally, in our analysis the population demographic structure was not explicitly included, and the model treats each NUTS 3 region as a single population unit. Since susceptibility to leptospirosis may vary by age, this simplification could lead to either over- or underestimation of incidence depending on regional age distributions. Finally, because the analysis relies on aggregated regional data, the potential for ecological fallacy remains, as regional patterns may not directly reflect individual-level exposures. Despite the inherent data constraints, we believe our findings offer valuable insights into leptospirosis risk across Europe and can inform future public health decision-making.

We constructed a set of model combinations incorporating climate and environmental covariates, namely SPEI-3 as a drought indicator, Tmean for temperature, the forest human nexus as a proxy for wildlife–human interaction and forest encroachment, and mammal richness as an indicator of biodiversity. The best-fitting model included all covariates. Our analysis showed that much of the seasonal variation in leptospirosis incidence rate could be explained by Tmean alone. Inter-annual variability was somewhat influenced by SPEI-3, reflecting the impact of notable climate anomalies in Europe during the study period. In 2022, the continent experienced record-breaking heat and one of the most severe droughts on record, with widespread soil moisture deficits and reduced river flows.^20^ Earlier multi-year droughts (2018–2020) also affected large areas of central and southern Europe.^21^ Such dry conditions are generally less favourable for *Leptospira* spp. survival, consistent with observations that leptospirosis cases are more common in wet than in dry environments.^22^ However, during droughts, water and food scarcity may drive rodents closer to human settlements, increasing contact rates and the likelihood of pathogen spillover.^23^ These climatic extremes may therefore have influenced environmental conditions and exposure pathways relevant to leptospirosis transmission, underlining the need to consider both climate and ecological factors in surveillance.

Our analysis revealed positive associations between climate factors and leptospirosis incidence rates. In particular, higher SPEI-3 and Tmean, each lagged by one month, were associated with an elevated risk of disease. The RR exhibited an approximately linear relationship with SPEI, whereas for Tmean it followed an exponential pattern. These associations likely reflect favourable conditions for rodent population growth and the persistence of *Leptospira* spp. in the environment.^24^ The inclusion of lagged variables demonstrates the temporal dimension of the exposure–response relationship, emphasising how climate conditions shape leptospirosis risk in the months preceding an outbreak. These findings highlight the potential value of climate-informed early warning systems for detecting anomalous or out-of-season events.

Our results are broadly consistent with previous research reporting positive associations between precipitation and leptospirosis risk,^9^, ^10^, ^11^^,^^25^ although in our analysis this relationship was represented indirectly through SPEI-3. Positive (high) SPEI values indicate wetter-than-normal conditions, which may result from above-average rainfall and/or reduced water loss, rather than precipitation alone. It should also be noted that many of these earlier studies were undertaken in tropical climates.^9^, ^10^, ^11^^,^^25^ By contrast, some investigations have found no correlation between precipitation and leptospirosis,^26^ while others have even linked outbreaks to drier conditions.^27^ For instance, in Israel, faecal contamination of water bodies by wild boar and cattle facilitated human infection under conditions of drought and water stagnation.^27^ These contrasting patterns are not unexpected, as drought has been associated with other disease outbreaks in Europe (e.g., plague).^28^ In fact, rainfall–rodent interactions are complex and context-dependent, with evidence suggesting that rodent responses to precipitation may be nonlinear; for example, extremely heavy rainfall can reduce rodent populations, potentially altering transmission dynamics.^29^

In addition to SPEI-3, Tmean also appears to influence leptospirosis risk. Consistent with our findings, numerous studies have reported positive associations between higher temperatures and increased incidence rate,^9^, ^10^, ^11^^,^^26^ highlighting the broader importance of climatic conditions in disease transmission. Higher temperatures may prolong the survival of *Leptospira* spp. in soil and water, though in vivo evidence is limited.^30^ Warmer conditions may also increase human exposure through water-based activities and support larger rat populations,^31^ further increasing the transmission risk. In general, leptospirosis in tropical regions is largely driven by heavy rainfall and flooding, while in high-income countries, including Europe, cases are increasingly linked to recreational water activities, although their exact contribution to overall incidence remains unclear.^3^ It is important to highlight that climate-driven changes in leptospirosis dynamics in tropical regions may also have indirect implications for Europe through travel, mobility, and changing reservoir distributions, although these pathways were beyond the scope of our analysis.

In contrast with the climate factors, we observed a negative relationship between mammal richness and leptospirosis incidence rate, consistent with the findings of Derne et al.^32^ These authors suggested that ecosystem disruption, including reducing biodiversity, may contribute to higher leptospirosis incidence rate in humans and found statistically significant evidence supporting this hypothesis. Our results similarly support the dilution effect, whereby increased mammal richness may limit disease transmission, potentially through the regulation of rat populations via direct competition or predation.^32^ However, it is important to highlight that leptospirosis risk may be driven by a few dominant rodent reservoirs, and increased richness does not guarantee reduced transmission. The role of biodiversity in emerging infectious disease risk remains debated,^33^ with effects often being nonlinear^34^ and context dependent. Due to the complexity of disease ecology, overall biodiversity does not straightforwardly predict risk; rather, the presence and interactions of specific host species, vectors, and pathogens appear to exert greater influence on local transmission.^33^ Thus, rather than indicating a true dilution mechanism, our findings likely capture broader ecological conditions, and interpretation should focus on the composition and dynamics of specific host communities rather than biodiversity per se.

Our analysis also highlights a complex relationship between the forest human nexus and leptospirosis incidence rates. Risk appears to increase when humans are closer to forests, but the association is nonlinear. A plausible explanation is that moderate forest–human interface levels increase opportunities for contact with rodent reservoirs typical of edge habitats, while very high values may correspond to more remote, sparsely populated forests where human exposure is limited, producing a plateau. In these latter settings, rodent reservoirs may be abundant, but opportunities for humans to encounter contaminated environments drop simply because fewer people live, work, or recreate in these deep-forest landscapes. In simple words, this may mean that the risk is highest at forest edges or peri-urban interfaces, not deep inside forests. However, because the index reflects broader landscape–human interaction rather than specific rodent ecology, it should be interpreted as a general land-use proxy, and mechanistic conclusions should be drawn with caution.

Anthropogenic activities, such as urbanisation and deforestation, can facilitate the expansion of rodent populations, while habitat destruction forces rodents to migrate, often bringing them closer to human settlements and domestic animals.^4^ Interestingly, Richardson et al.^35^ recently reported that rat populations increased most in cities experiencing greater warming, higher population density, and greater urbanisation. While the increases were observed across cities regardless of affluence (per capita GDP), the authors note that cities showing declining rat populations often implemented proactive pest control measures and maintained high sanitation standards, suggesting that effective management can mitigate these trends.^35^ In our study urbanisation was not included explicitly, but its effects are partially captured through the forest human nexus and mammal richness, as studies have documented that urbanization can alter the spatial distribution of forest-adjacent communities,^14^ with some regions experiencing migration away from forests and others increasing settlement near forest edges, while lower species diversity often reflects highly urbanised areas.^36^ Overall, changes in land use, beyond forest alteration, are likely to reshape transmission patterns of rodent-borne diseases.^37^^,^^38^ These shifts can alter host populations, reorganise wildlife communities, and modify host–pathogen interactions, often increasing pathogen prevalence in species that thrive in disturbed environments.^37^ Evidence from a meta-analysis examining rodent responses to changes in land use further indicates that such transformations may elevate the risk of zoonotic pathogen transmission.^37^ However, rodents are not the only reservoirs of *Leptospira* spp.; livestock and pets may also play an important role, reflecting the ecological flexibility of *Leptospira* spp. and its adaptability to multiple host species.^1^ This complexity emphasises the challenges of predicting leptospirosis risk and underscores the need to account for diverse ecological and environmental contexts in public health planning.

Regarding the spatial and temporal patterns of the disease, our model predicts that leptospirosis incidence rate peaks in August and September, particularly in warm coastal regions where elevated temperatures and higher-than-average rainfall create conditions favourable for transmission. Several densely populated areas are also projected to experience higher incidence rates, possibly due to reduced mammal richness and its effect on pathogen–host dynamics. Overall, the uncertainty around the incidence rate remains low; however, it increases in certain regions and during the winter months (see Supplementary Material), suggesting that local environmental or socio-economic factors may influence disease dynamics and warrant further investigation.

Reservoirs are central to leptospirosis risk, with rodents as the main hosts of *Leptospira* spp.^1^ A new dataset on the spatial distributions of rodent genera combining GBIF records with climate and environmental variables became available during our study, offering projections under four future climate scenarios.^6^ However, its temporal resolution was incompatible with our analysis. Our model relies on climate and environmental variables as proxies for both reservoir dynamics and human exposure. These factors strongly influence rodent distribution and population dynamics by shaping habitat suitability, food availability, and biotic interactions.^4^ At the same time, favourable weather conditions increase human outdoor activity, thereby heightening transmission risk.^39^ Considering this, although a simplification of multiple interconnected risk factors, our approach provides a robust basis for capturing the spatial and temporal patterns of leptospirosis risk.

Other hosts, such as livestock, also play a role in the transmission of *Leptospira* spp. to humans.^1^ A key limitation of this study is the absence of animal leptospirosis data, as systematic surveillance is limited since the disease is no longer on the WOAH list.^12^ This constraint prevents the inclusion of animal infection data and hinders a comprehensive One Health assessment. With regard to the inclusion of other animal information, although the FAO gridded global livestock density dataset is available,^40^ it was unsuitable for our analysis because management practices and biosecurity measures are likely more influential than density alone.^41^ This information is generally collected by the individual farms and is not publicly accessible, making it unavailable at the NUTS 3 scale. Farm structure information is also available from EUROSTAT at the NUTS 2 level,^42^ but many regions lack official data, preventing its inclusion in our analysis. In particular, the number and distribution of small-scale farms, which typically operate with lower biosecurity standards and may therefore represent a higher risk for pathogens such as *Leptospira* spp., especially in pigs and cattle, cannot be resolved at the required spatial scale using currently available datasets.

Similarly, covariates related to recreation, flood exposure, and infrastructure would have been valuable in the model, but consistent European-wide data are lacking. In particular, for flood risk, most available products are based on climate simulations rather than observed events, while satellite-derived datasets and records of observed flood events^43^ contain too few events in our study area and our reference period to support robust modelling. More recent databases, such as HANZE, provide improved coverage at the European level but extend only to 2020 and remain spatially biased across countries.^44^ We therefore excluded flood risk as a covariate, as these data are still insufficient for integration at the required resolution.

Our scenario analysis allowed climate covariates (SPEI-3 and Tmean) to vary according to projected climate scenarios, while non-climate factors were held constant at current levels. This key assumption reflects a scenario in which only climate changes over time, whereas human population, land use and host community composition remain unchanged. Under these assumptions, our projections indicate that leptospirosis incidence rates in Europe are likely to increase, with greater magnitude under high GHG emission scenarios (SSP5-8.5) compared with more moderate pathways (SSP2-4.5). The divergence between scenarios becomes increasingly pronounced over the long-term period (2081–2100), highlighting the sensitivity of future disease risks to GHG emission trajectories. Although small areas may experience decreases, these are rare compared with the widespread projected increases.

In the future scenarios, an increasing number of NUTS 3 are projected to experience statistically significant rises in disease incidence rate, particularly during months that historically showed lower activity (e.g., autumn and winter). This suggests that climate conditions will become progressively more favorable for disease transmission throughout a larger part of the year. As temperatures increase, regions and seasons that were previously too cool, particularly in northern and central Europe, may become more conducive to pathogen survival and transmission, leading to a relative expansion of risk outside the summer months. However, it is worth noting that despite statistically significant increases were detected in some areas, the absolute incidence may remain small in magnitude, especially in historically low-incidence northern Europe.

The projected decreases in incidence, such as those observed in Turkey during autumn under all scenarios and sporadically in parts of Spain and the United Kingdom, suggest region-specific mechanisms. In southern Europe, including Turkey and parts of Spain, progressive warming and drying may reduce the persistence of *Leptospira* spp. in the environment, as high temperatures and low moisture limit bacterial survival. Conversely, declines in the United Kingdom and other temperate regions may indicate the influence of other factors, such as socio-economic changes, that modulate exposure risk independently of climate. These heterogeneous patterns highlight the nonlinear response of leptospirosis dynamics to climate forcing and underscore the importance of considering both climatic thresholds and local contextual factors in projecting future disease risk. Nevertheless, it is important to note that the overall seasonal pattern remains stable: the highest estimated incidence rate is still expected to occur in August and September, consistent with the reference period (see Supplementary Material). This indicates that, although the transmission window may expand, the fundamental timing of peak incidence rate will not shift.

These findings carry important implications for the European Union, confirming the importance of its ambitious climate policy, i.e. the European Green Deal and Fit-for-55 targets. At the same time, public health systems must adapt by strengthening disease surveillance for leptospirosis and by integrating climate and health modelling into early warning systems, supporting prompt public health action. Environmental and urban measures will also be critical, including improved flood and water management, enhanced urban drainage and sanitation, and more effective rodent control. Finally, greater investment in research on climate-sensitive infectious diseases, together with awareness campaigns in regions at heightened risk, will be essential for building resilience against the growing threat of leptospirosis under climate change.

This concern also extends beyond leptospirosis and Europe. It is estimated that over half of all known human pathogenic diseases may be exacerbated by climate change through a wide range of climatic hazards triggered by ongoing GHG emissions, including 58% of all infectious diseases recorded in human history.^5^ The large number of affected pathogens and transmission pathways highlights the scale of the threat to human health and the urgent need for decisive action to mitigate GHG emissions.

A comprehensive vulnerability assessment is essential to evaluate the ability of communities to cope with leptospirosis, taking into account susceptibility, exposure, preparedness, and responsiveness. Ideally, this would include healthcare infrastructure and socio-economic indicators, though data limitations may restrict full-scale analyses. In this study, healthcare system vulnerability is approximated only through hospital bed availability, which is a clear limitation, as it does not capture primary care capacity, diagnostic readiness, veterinary and environmental health services, or other sectors critical for zoonotic disease prevention. Our simplified assessment suggests that countries with fewer hospital beds are characterised by a higher leptospirosis incidence rate. It is important to note that, although the disease is often asymptomatic, it can cause severe illness, including jaundice and multi-organ dysfunction, with an estimated one million cases and ∼60,000 deaths globally each year.^1^ According to the latest ECDC annual report (2022), approximately 42% of all confirmed cases required hospitalization, underscoring the substantial clinical burden of the disease.^45^ Some patients experience prolonged illness and severe sequelae,^1^ highlighting that the burden of leptospirosis cannot be overlooked, particularly under climate change and rising incidence rates in Europe.

### Conclusions

Our regional study shows that higher temperatures and wetter conditions elevate leptospirosis risk, reinforcing the evidence that the disease is climate-sensitive in Europe. Lower risk observed in areas with reduced mammal richness suggests that greater biodiversity may help limit transmission, while the complex, nonlinear relationship between human proximity to forests and disease incidence highlights the multifaceted nature of exposure.

According to our projections, leptospirosis incidence in Europe is expected to increase in both the short-term (2041–2060) and long-term (2081–2100) under the SSP2–4.5 and SSP5–8.5 climate scenarios. This highlights opportunities for proactive intervention: reducing GHG emissions and limiting climate change will be essential to mitigating future disease risks. Integrating spatiotemporal models into climate adaptation strategies and disease early warning systems can further enhance outbreak forecasting and enable more timely and targeted public health responses.

Given the zoonotic nature of leptospirosis, coordinated One Health surveillance across humans, animals, and the environment is crucial to support these efforts. Monitoring animal hosts and environmental reservoirs can provide essential data for predictive models, inform interventions, and strengthen public health resilience against climate-sensitive diseases such as leptospirosis. Through environmental management, climate mitigation, and predictive disease modelling, we can reduce the burden of climate-sensitive diseases such as leptospirosis and strengthen public health resilience.

## Contributors

AF conceptualised and designed the study. BA, AD and RL helped AF with the methodology. AF had access to and verified the data reported in the manuscript and undertook data analysis. AF drafted the manuscript. JCC and WS supervised the study and had final responsibility for the decision to submit for publication. All authors contributed to review and editing.

## Data sharing statement

Human cases data are available upon request from *The European Surveillance System (TESSy)* (https://www.ecdc.europa.eu/en/publications-data/european-surveillance-system-tessy). Monthly temperature data are available from the Copernicus Climate Data Store ERA5 (https://cds.climate.copernicus.eu/datasets/reanalysis-era5-single-levels-monthly-means). Forest human nexus data can be obtained following the code of Massaro et al.,^14^ accessible on GitHub (https://github.com/emanuelemassaro/Forest_Human_Nexus). Mammal species richness data are available from the IUCN Red List Spatial Dataset (https://www.iucnredlist.org/resources/spatial-data-download) following the approach developed by the Knowledge Center for Biodiversity-Global Biodiversity Data (KCBD-GBD; ex-DOPA) unit of the JRC.^15^ Population data are available from the Global Human Settlement Layer (GHSL) (https://human-settlement.emergency.copernicus.eu/). SPEI-3 data are available from the SPEI database (SPEIbase v.2.10) developed by the Climatology and Climate Services Laboratory (https://digital.csic.es/handle/10261/364137). Future climate projection data from the NEX-GDDP-CMIP6 dataset are available from NASA's Socioeconomic Data and Applications Center (SEDAC) for SPEI (https://www.ciesin.columbia.edu/data/globaldrought/) and from the NASA Center for Climate Simulation (NCCS) for temperature (https://www.nccs.nasa.gov/services/data-collections/land-based-products/nex-gddp). The number of hospital beds per country is available from EUROSTAT (https://ec.europa.eu/eurostat/databrowser/view/HLTH_RS_BDS1/default/table?lang=en). An example dataset and sample code to reproduce the analysis are available at https://github.com/anjelinejeline/leptospirosis_europe_sim.

## Editor note

The Lancet Group takes a neutral position with respect to territorial claims in published maps and institutional affiliations.

## Declaration of interests

No authors have any relationships or activities that could appear to have influenced the submitted work. The views expressed in this article are purely those of the authors and may not, under any circumstances, be regarded as an official position of the European Commission.
