# Supplementary material for: Spatiotemporal dynamics of leptospirosis in Europe: a retrospective observational study with prospective projections
Source: Lancet Reg Health Eur. 2026 Apr 6;65:101671. doi: 10.1016/j.lanepe.2026.101671 (PMC13090733; doi:10.1016/j.lanepe.2026.101671)
Supplement: Supplementary Material [file mmc1.docx]

**Spatiotemporal dynamics of leptospirosis in Europe: a retrospective observational study with prospective projections**

Angela Fanelli, PhD ^a,b*^ (ORCID: 0000-0002-8204-1230), Baptiste Alglave, PhD^c^, Luca Caporaso, PhD^d,e^, Alessandro Cescatti PhD^d^, Juan-Carlos Ciscar, PhD^a^, Gregoire Dubois PhD^d^, Alessandro Dosio PhD^d^, Rosa M Estevez-Reboredo PhD^f,g^ (ORCID: 0000-0001-5241-9725), María Blazquez, MPH^h^ (ORCID: 0000-0002-3069-4029), Dolores Ibarreta PhD^a^, , Andrea Mandrici PhD^i^, Emanuele Massaro, PhD^d,^ Prof Rachel Lowe, PhD^j,k,l,^ and Wojtek Szewczyk, PhD^a^.

a. European Commission, Joint Research Centre (JRC), Seville, Spain

b. Facultad de Veterinaria, Universidad Alfonso X el Sabio (UAX), Avenida de la Universidad 1, 28691 Villanueva de la Cañada, Madrid, España.

c. Université Bretagne Sud, Lab-STICC, 56000, Vannes, France.

d. European Commission, Joint Research Centre (JRC), Ispra, Italy.

e. National Research Council of Italy, Institute of BioEconomy, Rome, Italy

f. Centro Nacional de Epidemiología, Instituto de Salud Carlos III (ISCIII), Madrid, Spain.

g. Consortium for Biomedical Research in Epidemiology and Public Health (CIBERESP), Madrid, Spain.

h. Escuela Nacional de Sanidad, Instituto de Salud Carlos III (ISCIII), Madrid, Spain.

i. Arcadia SIT S.r.l., 27029 Vigevano, Italy.

j. Barcelona Supercomputing Center (BSC), Barcelona, Spain.

k. Catalan Institution for Research and Advanced Studies (ICREA), Barcelona, Spain.

l. Centre on Climate Change and Planetary Health and Centre for Mathematical Modelling of Infectious Diseases, London School of Hygiene and Tropical Medicine, London, United Kingdom.

** Corresponding author: European Commission, Joint Research Centre (JRC), Seville, Spain. E-mail address:* [*Angela.FANELLI@ec.europa.eu*](mailto:Angela.FANELLI@ec.europa.eu)

***Supplementary material***

Temporal variation in leptospirosis cases between January 2010 and December 2023 is shown in Figure S1 and S2.

**Figure S1: Monthly leptospirosis cases between January 2010 and December 2023**

**
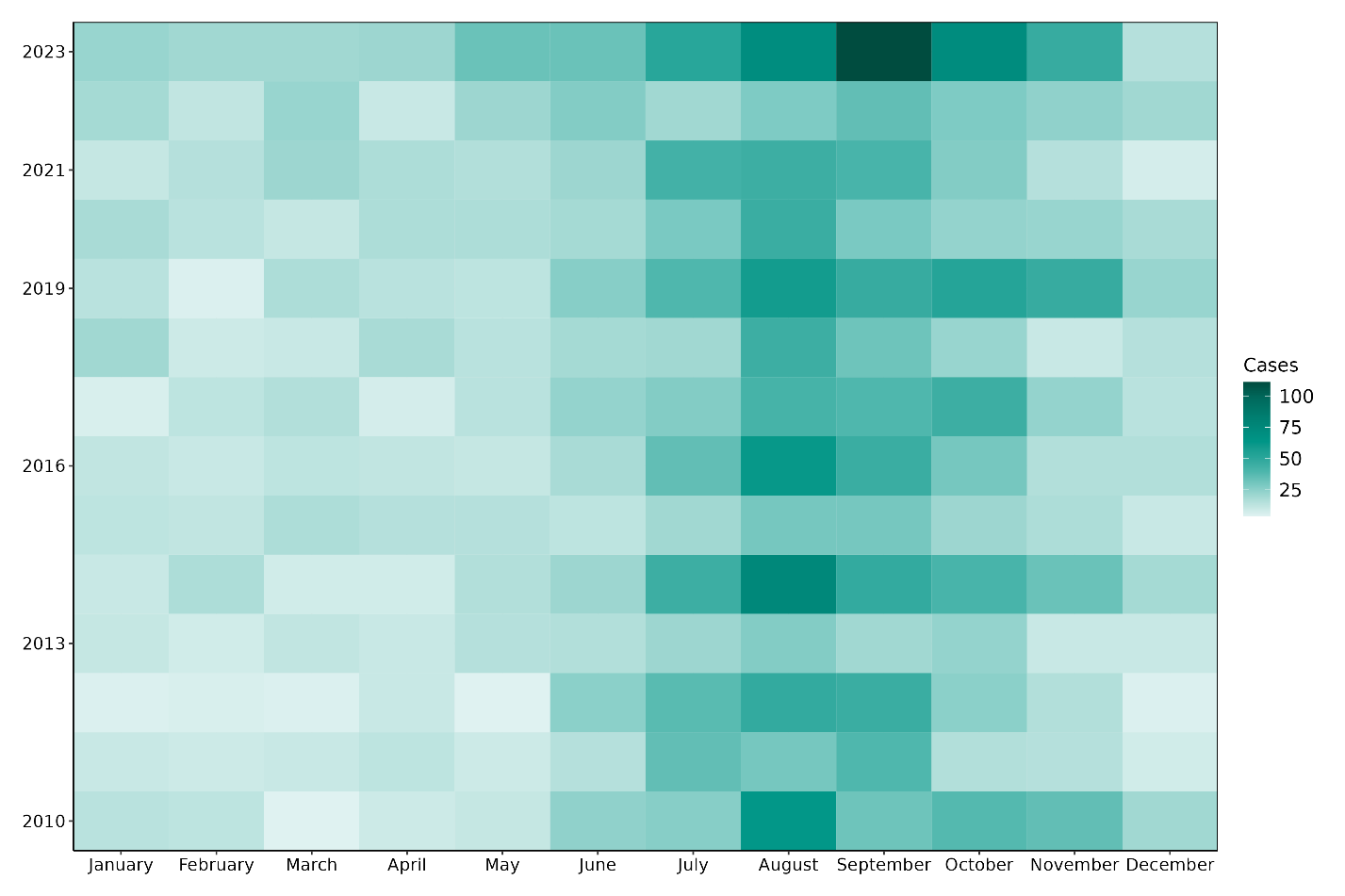
**

**Figure S2: Total leptospirosis cases per month, 2010-2023**

**
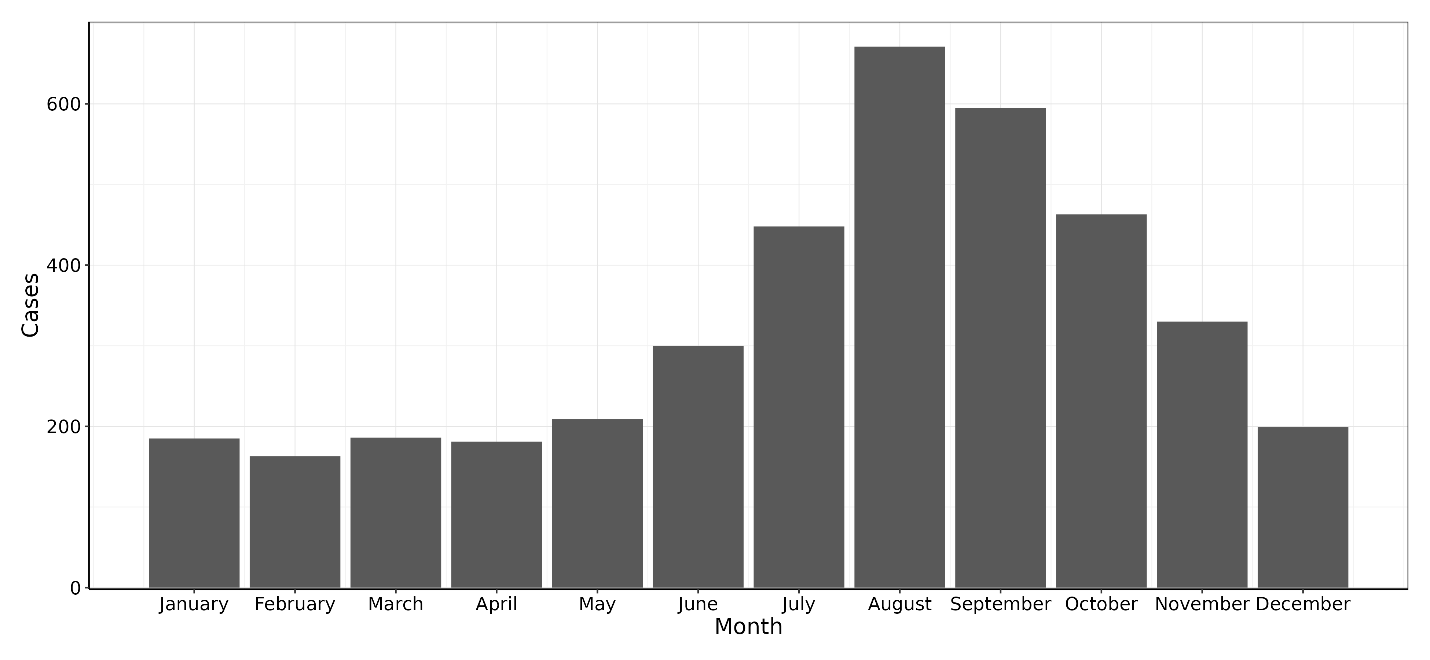
**

Figure S3 shows the annual trends of total cases (A) and the number of reporting NUTS 3 regions (B).

**Figure S3. Annual leptospirosis burden and NUTS 3 reporting the disease across the EU, 2010–2023. (A) Total number of reported leptospirosis cases per year aggregated across all EU NUTS 3 regions. (B) Number of NUTS 3 reporting at least one leptospirosis case per year.**

**
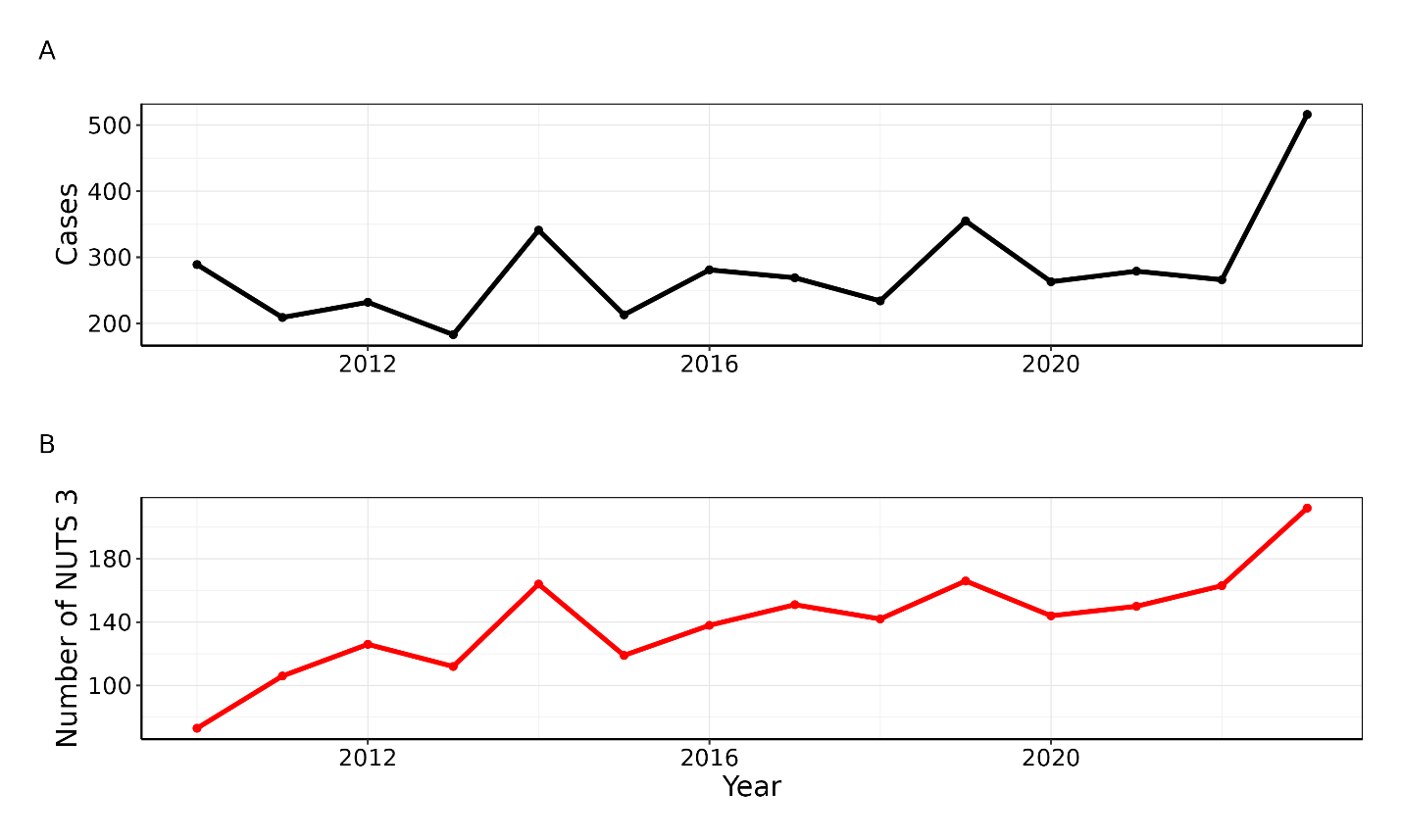
**

Figure S4 shows the correlation matrix of the covariates considering Pearson’s correlation coefficients. Covariates included mean temperature lagged by 1 month (denoted *t2m_lag* in Figure S4), 3-month Standardised Precipitation–Evapotranspiration Index (SPEI-3) lagged by 1 month (denoted *spei_lag* in Figure S4), forest human nexus (denoted *fhn* in Figure S4), and mammal richness (denoted *mammal_richness* in Figure S4).

**Figure S4: Correlation matrix of the covariates**


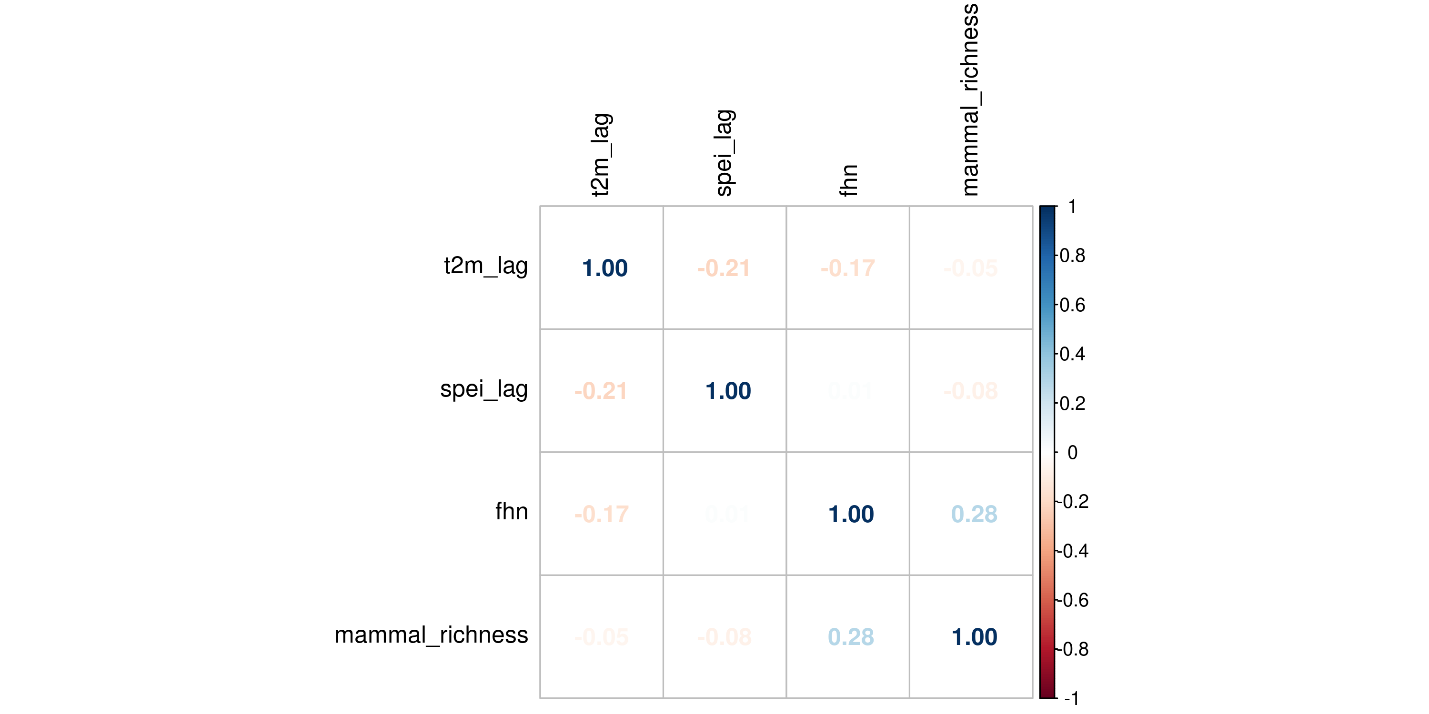


**Statistical model development**

Table S1 summarises all model combinations and their evaluation metrics. All models included an intercept and at least one random effect: monthly autocorrelated effects for seasonality (first-order random walk, denoted *m* in Table S1), year-level effects for inter-annual variability (independent Gaussian random effect, denoted *y* in Table S1), and NUTS 3–level spatial effects (modified Besag–York–Mollié model, denoted *NUTS 3* in Table S1). Covariates (second-order random walks) included mean temperature lagged by 1 month (denoted *Tmean* in Table S1), SPEI-3 lagged by 1 month (*SPEI-3* in Table S1), forest human nexus (denoted *FHN* in Table S1), and mammal richness (denoted *MR* in Table S1). The evaluation metrics considered were the Deviance Information Criterion (*DIC* in Table S1), the effective number of parameters (*P.eff* in Table S1), the mean cross-validated log score (*Log score* in Table S1), and the CPO, a leave-one-out predictive measure of fit. For the latter we calculated the number of observations for which CPO calculation failed (*CPO fail* in Table S1).

**Table S1: List of models developed and relative metrics**

| Model | Random effects | DIC | P.eff | Log  score | CPO fail |
| --- | --- | --- | --- | --- | --- |
| Model 15 | m + NUTS 3+ y + Tmean + SPEI-3 + FHN +MR | 20993.31 | 254.9198 | 0.445014 | 0 |
| Model 12 | m + NUTS 3+ y + Tmean + SPEI-3+ MR | 20994.96 | 259.0715 | 0.445049 | 0 |
| Model 11 | m + NUTS 3+ y + Tmean + SPEI-3+ FHN | 21002.17 | 260.9886 | 0.445213 | 0 |
| Model 5 | m + NUTS 3+ y + Tmean + SPEI-3 | 21003.89 | 264.027 | 0.445247 | 0 |
| Model 13 | m + NUTS 3+ y + Tmean + FHN + MR | 21047.12 | 253.947 | 0.446156 | 0 |
| Model 7 | m + NUTS 3+ y + Tmean + MR | 21049.19 | 258.1295 | 0.446199 | 0 |
| Model 14 | m + NUTS 3+ y + SPEI-3+ FHN + MR | 21049.71 | 245.7396 | 0.446213 | 0 |
| Model 9 | m + NUTS 3+ y + SPEI-3+ MR | 21051.26 | 249.5826 | 0.446245 | 0 |
| Model 6 | m + NUTS 3+ y + Tmean + FHN | 21055.86 | 260.152 | 0.446355 | 0 |
| Model 1 | m + NUTS 3+ y + Tmean | 21057.56 | 263.1186 | 0.446389 | 0 |
| Model 8 | m + NUTS 3+ y + SPEI-3+ FHN | 21063.23 | 255.2039 | 0.446511 | 0 |
| Model 2 | m + NUTS 3+ y + SPEI-3 | 21064.13 | 257.2836 | 0.446528 | 0 |
| Model 10 | m + NUTS 3+ y + FHN + MR | 21097.78 | 244.0433 | 0.447235 | 0 |
| Model 4 | m + NUTS 3+ y + MR | 21099.68 | 247.9172 | 0.447272 | 0 |
| Model 3 | m + NUTS 3+ y + FHN | 21110.26 | 253.1643 | 0.447516 | 0 |
| Base model | m + NUTS 3+ y | 21111.87 | 255.3593 | 0.447544 | 0 |
| Model with only the seasonal effect | m | 22386.26 | 14.46842 | 0.474504 | 0 |
| Model with only the annual effect | y | 23049.18 | 15.91501 | 0.488552 | 0 |

Figure S5 displays the monthly random effects representing seasonality for models used to evaluate the contribution of climate covariates.

**Figure S5: Monthly random effects from a model including only seasonality (bright pink), the best model (teal green), the best model excluding SPEI-3 (purple) and the best model excluding Tmean (orange)**

**
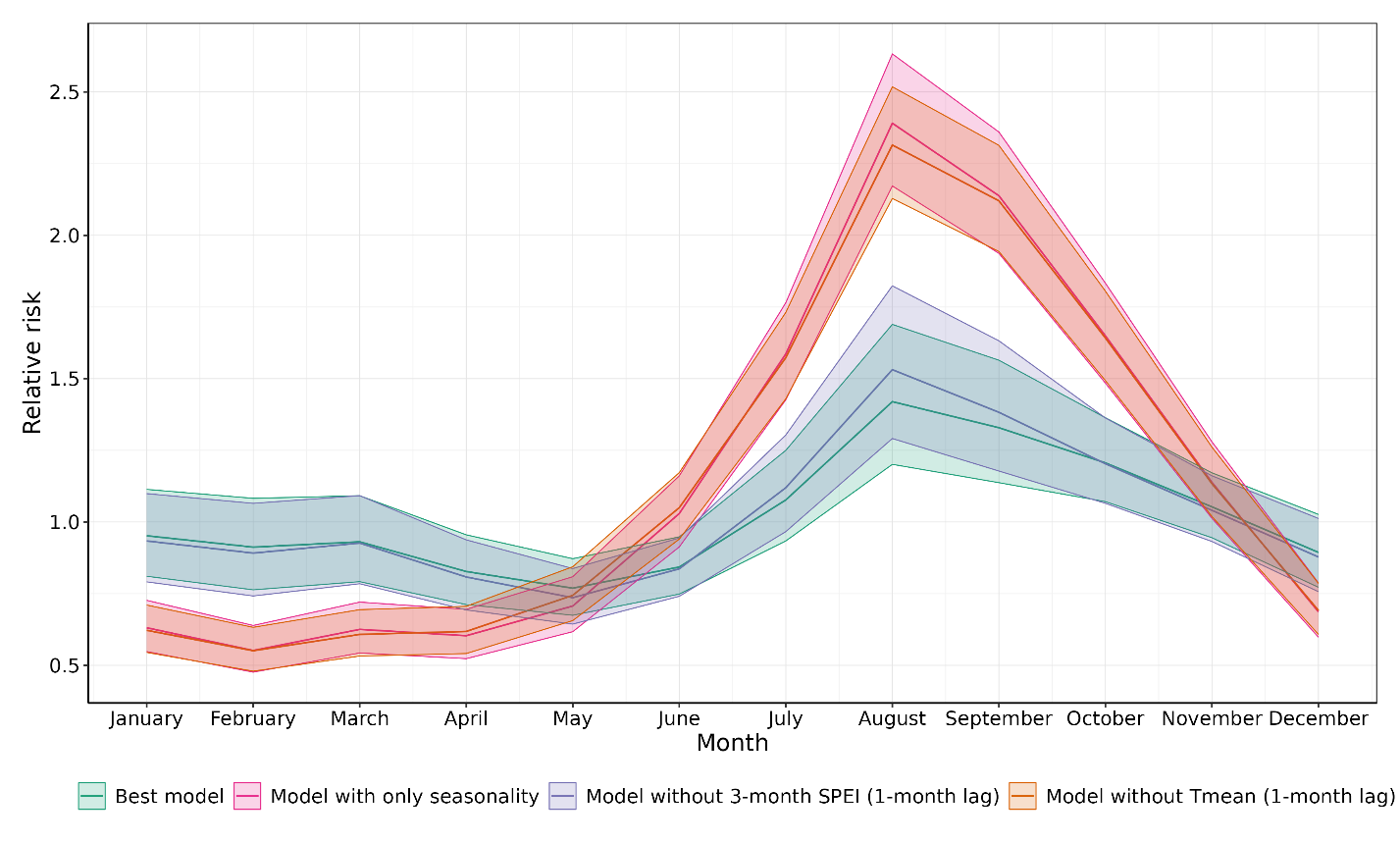
**

Figure S6 displays the yearly random effects representing inter-annual variability for models used to evaluate the contribution of covariates. In some years (e.g., 2010, 2014, 2022) the inclusion of SPEI-3 in the model decreased the contribution of the yearly random effects in accounting for inter-annual variability.

**Figure S6: Yearly random effects from a model including only these effects (bright pink), the best model (teal green) and the best model excluding SPEI-3 (purple), the forest human nexus (olive green), and Tmean (orange)**

**
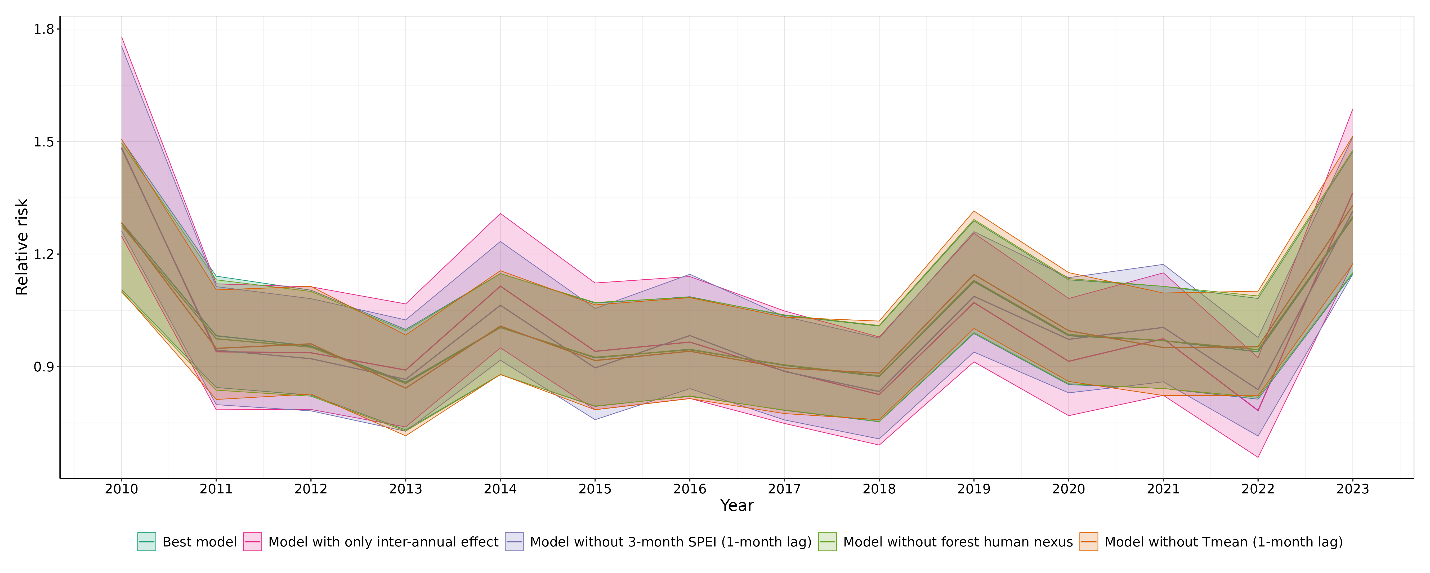
**

**Analysis of the best-fitted model**

Figures S7 and S8 illustrate the uncertainty of the best-fitted model, presented as the standard deviation (SD) and coefficient of variation (CV), respectively.

**Figure S7: Model uncertainty across NUTS 3 regions, expressed as the standard deviation of predicted incidence (reference period, 210-2023)**

**
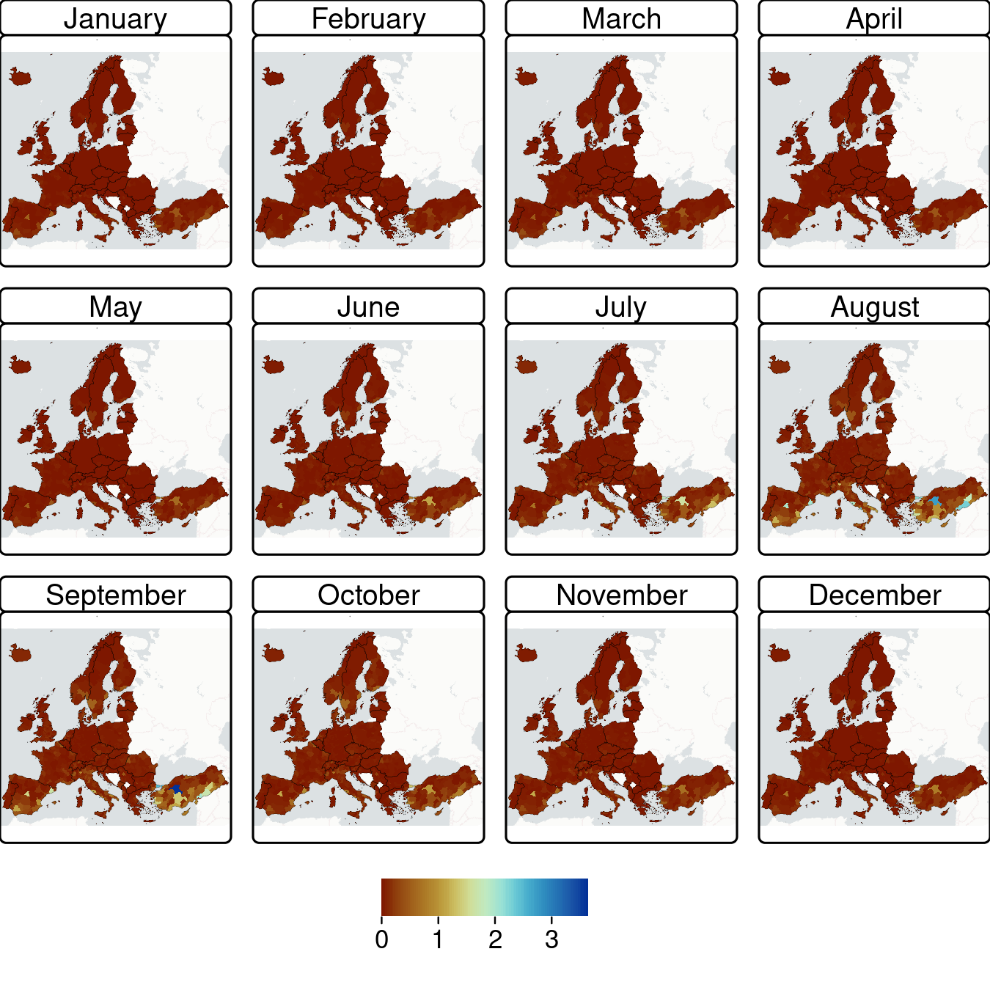
**

**Figure S8: Model uncertainty across NUTS 3 regions, expressed as the coefficient of variation of predicted incidence (reference period, 2010-2023)**


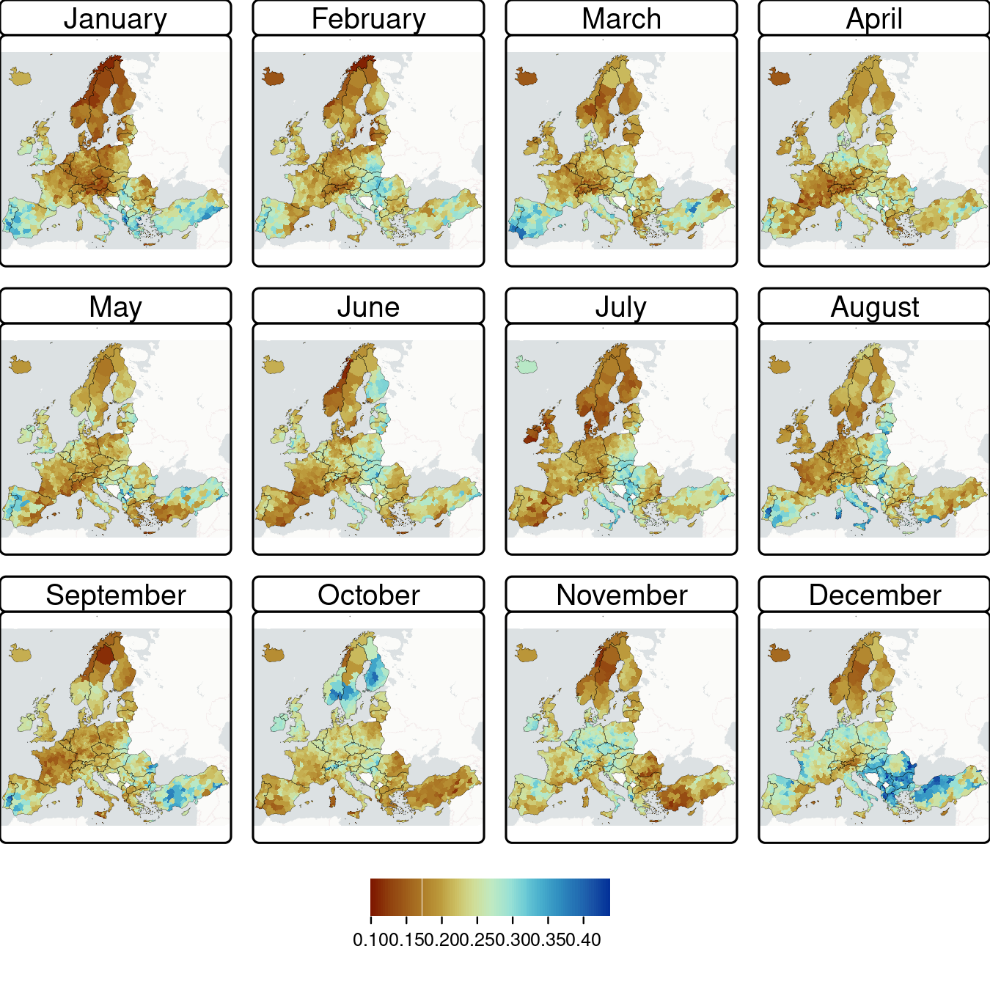


Figures S9 and S10 show the geographical distribution of risk and the total population by risk class and month, respectively. The total population at risk was calculated by averaging the population for each NUTS 3 and then summing the populations of all regions within each risk category.

**Figure S9: Spatial distribution of leptospirosis risk across Europe at the NUTS 3 level (reference period, 2010-2023)**

**
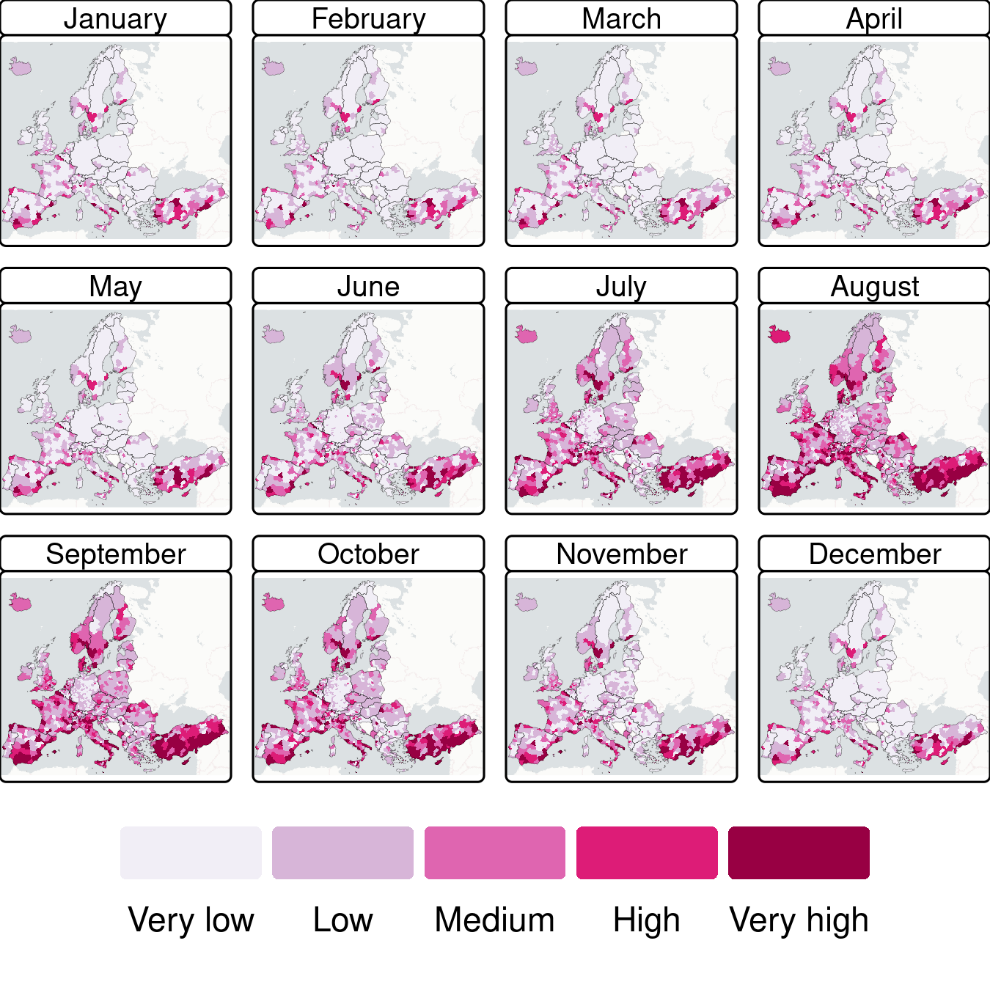
**

**Figure S10: Total population at risk per risk class and month, calculated by summing average populations of NUTS 3 regions within each risk category (reference period, 2010-2023)**

**
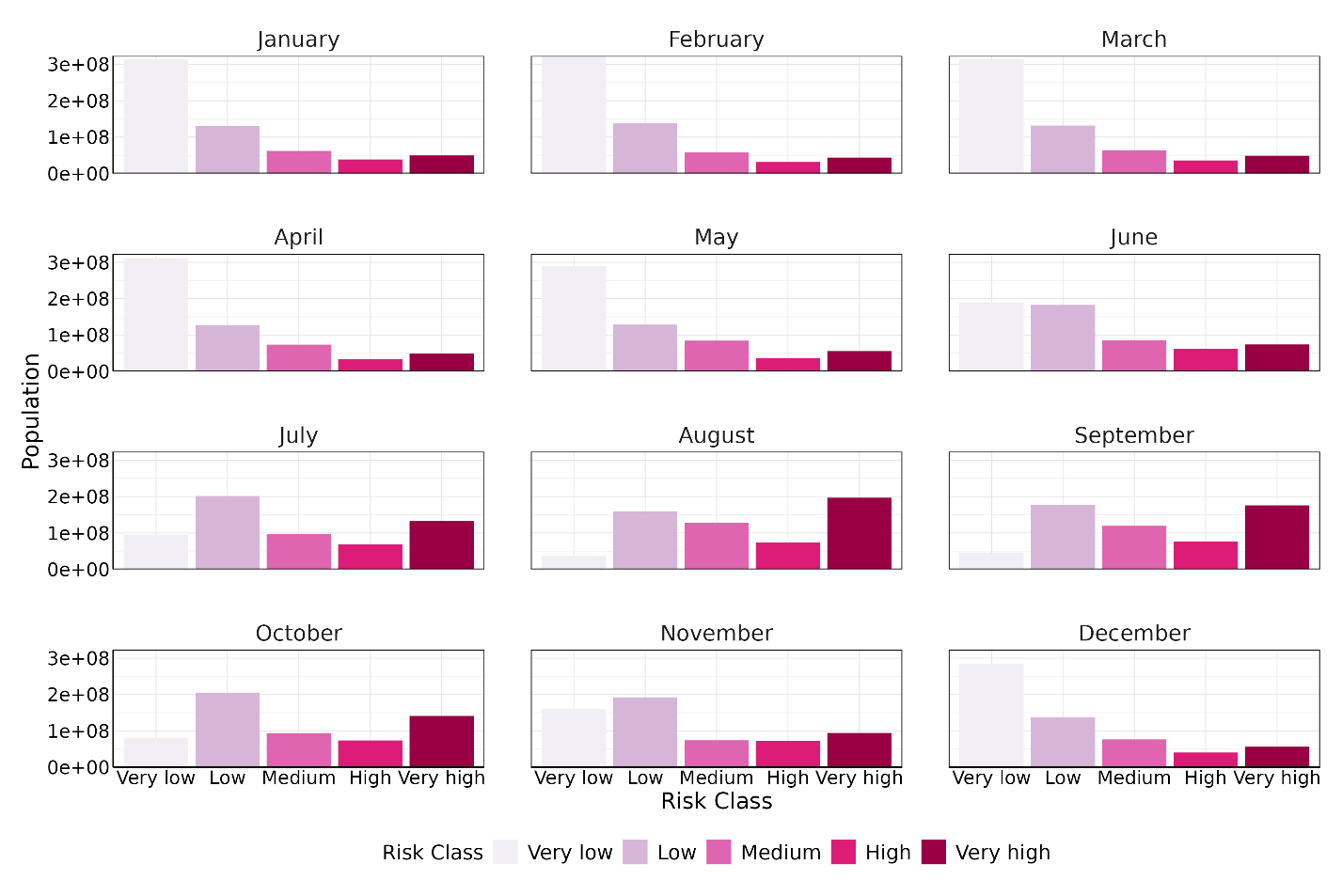
**

**Examining the impact of climate change under future scenarios**

To assess the potential impact of climate change on leptospirosis incidence, we quantified changes in monthly climate-driven incidence between a historical-reference period and future projections under the shared socioeconomic pathways SSP2-4.5 and SSP5-8.5, using a selected set of NEX-GDDP models. Future climate projection data from the NEX-GDDP-CMIP6 dataset are available from NASA’s Socioeconomic Data and Applications Center (SEDAC) for SPEI (https://www.ciesin.columbia.edu/data/globaldrought/) and from the NASA Center for Climate Simulation (NCCS) for Tmean (<https://www.nccs.nasa.gov/services/data-collections/land-based-products/nex-gddp>). The subset of models was selected to capture high climate variability. Model selection was conducted under the SSP5-8.5 scenario, as it shows the largest deviation from the historical period. For both variables, we calculated the annual mean over Europe for each year, considering three 20-year windows: 1995–2014 for the historical period, 2041–2060 for the short-term future, and 2081–2100 for the long-term future (Figure S11 and S12).

**Figure S11: Annual mean SPEI-3 across Europe for 20 NEX-GDDP models, shown for the historical period (1995-2014) and projected short-term (2041-2060) and long-term (2081-2100) future periods**


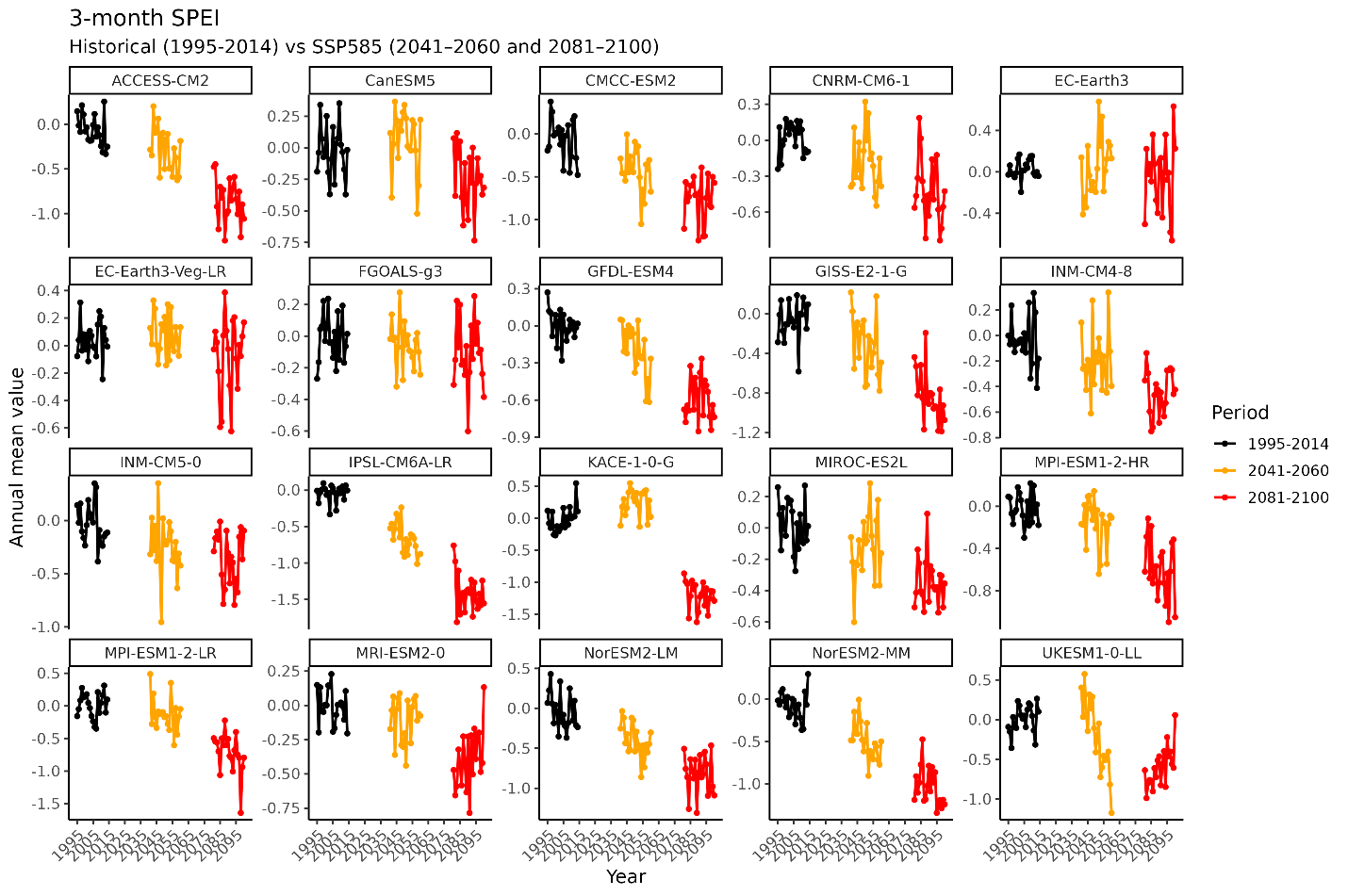


**Figure S12: Annual Tmean across Europe for 20 NEX-GDDP models, shown for the historical period (1995-2014) and projected short-term (2041-2060) and long-term (2081-2100) future periods**


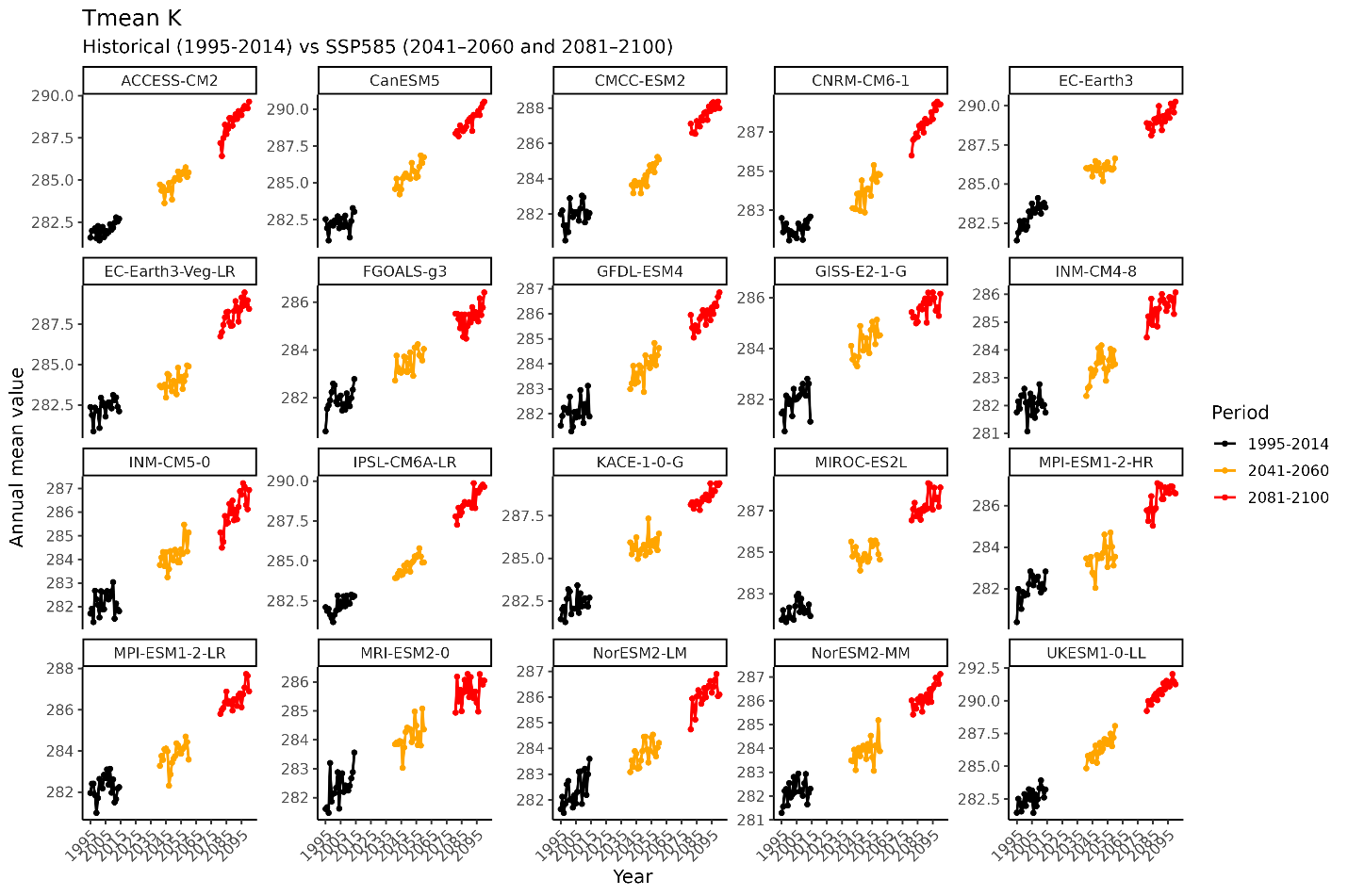


We then averaged values by period and computed the differences between the future periods and the historical baseline. Interannual variability is higher in the short-term future, reducing the signal strength; therefore, the long-term future was used for model selection. To represent the spread among models, we plotted the changes in SPEI-3 against Tmean for both short- and long-term futures (Figures S13 and S14) and selected five representative climate models based on their long-term characteristics. FGOALS-g3 was chosen as one of the driest and coldest models, IPSL-CM6A-LR as the driest model overall, CanESM5 as one of the warmest and least dry models, NorESM2-MM as both dry and cold, and CMCC-ESM2 to represent intermediate conditions.

**Figure S13: Differences (Δ) of SPEI-3 and Tmean between the short-term future (2041–2060) and the historical period (1995–2014) across 20 NEX-GDDP models, showing the spread among models**


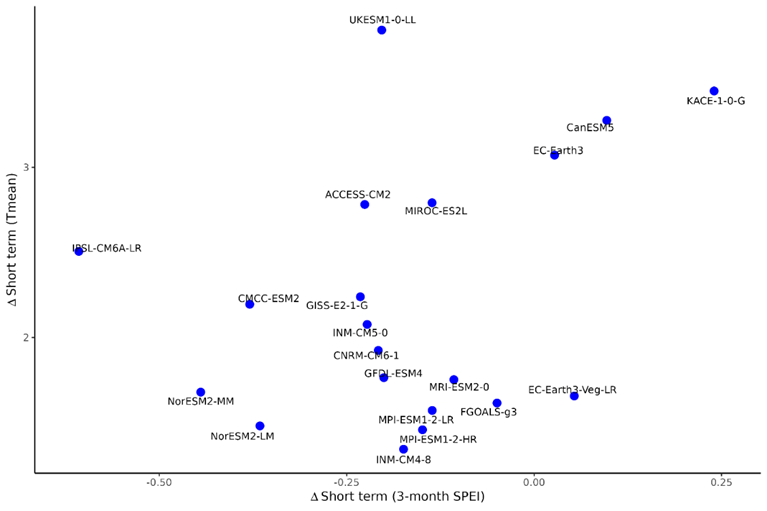


**Figure S14: Differences (Δ) of SPEI-3 and Tmean between the long-term future (2081–2100) and the historical period (1995–2014) across 20 NEX-GDDP models, showing the spread among models**


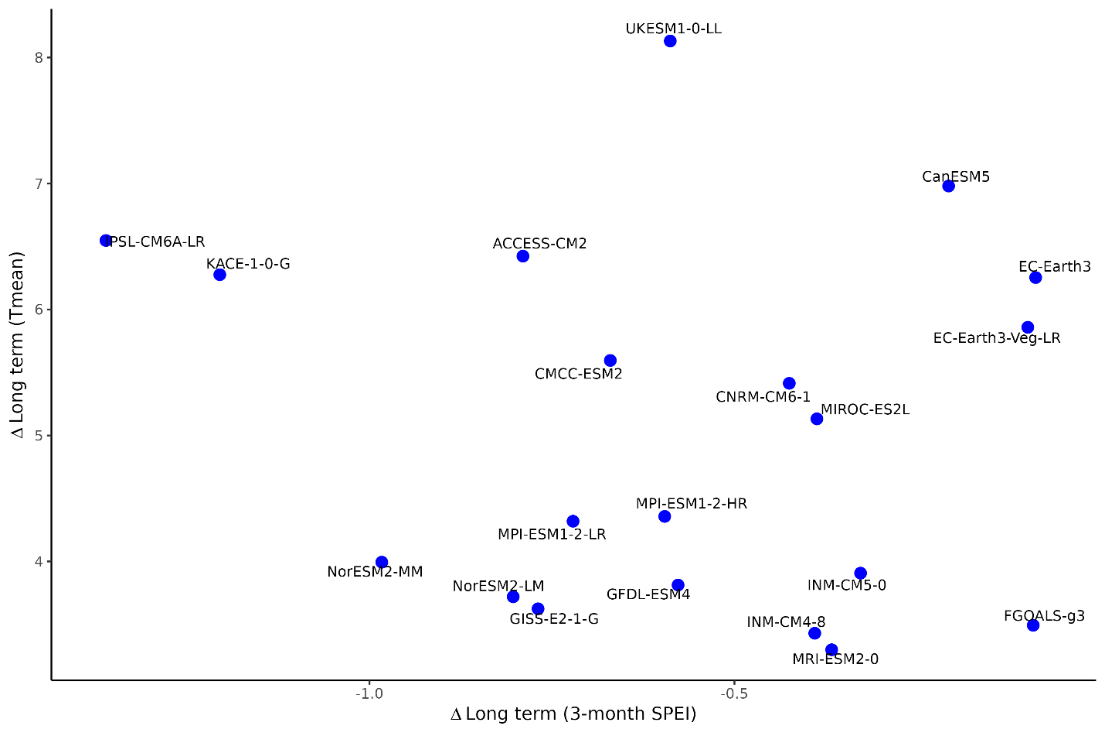


**Comparison of predictions using observed vs. NEX-GDDP inputs**

Before running future scenarios, we validated our approach by comparing predictions from the best-performing model for leptospirosis incidence using two alternative climate inputs:

1. **Observed datasets**:
   - SPEI-3 from the SPEI database (SPEIbase v.2.10) developed by the Climatology and Climate Services Laboratory *(*[*https://global-drought-crops.csic.es/*](https://global-drought-crops.csic.es/?utm_source=chatgpt.com)*)*,
   - Tmean from the Copernicus Climate Data Store ERA5 *(*[*https://cds.climate.copernicus.eu/datasets/reanalysis-era5-single-levels-monthly-means*](https://cds.climate.copernicus.eu/datasets/reanalysis-era5-single-levels-monthly-means) *)*.
2. **NEX-GDDP inputs**:
   - The five selected climate models (CanESM5, CMCC-ESM2, FGOALS-g3, IPSL-CM6A-LR, NorESM2-MM) covering the same reference period (2010–2023).
   - Historical simulations were used up to 2014, and extended to 2023 with SSP126, chosen because of its minimal divergence from observed trends.

This comparison ensured that predictions derived from NEX-GDDP inputs were consistent with those based on observed climate data before running future scenarios.

Results showed very high correlations between predictions using ERA5 + LCSC and those using NEX-GDDP models (r values: CanESM5: 0.97, CMCC-ESM2: 0.97, FGOALS-g3: 0.97, IPSL-CM6A-LR: 0.97, NorESM2-MM: 0.96). On average, all models slightly overestimated incidence relative to the observed-based predictions. Specifically, CanESM5 predicts 0.0025 cases per 100,000 people more than observed, CMCC-ESM2 predicts 0.0078 cases more, FGOALS-g3 predicts 0.0027 cases more, IPSL-CM6A-LR predicts 0.0027 cases more, and NorESM2-MM predicts 0.0031 cases more. These differences are negligible, amounting to less than one additional case per 100,000 people, indicating that all models provide predictions that are highly consistent with observed incidence. Despite this minor overestimation, these results confirm that NEX-GDDP-based predictions are highly consistent with those obtained from observed climate data. Figure S15 compares predictions of leptospirosis incidence using observed climate inputs with those obtained from the selected NEX-GDDP models for a random sample of 100 records. Figure S16 presents the time series of predicted incidence for each model, shown for a random sample of four NUTS 3 regions.

**Figure S15: Comparison of leptospirosis incidence predictions using observed climate data versus NEX-GDDP model inputs for 100 randomly selected records (reference period, 2010-2023)**


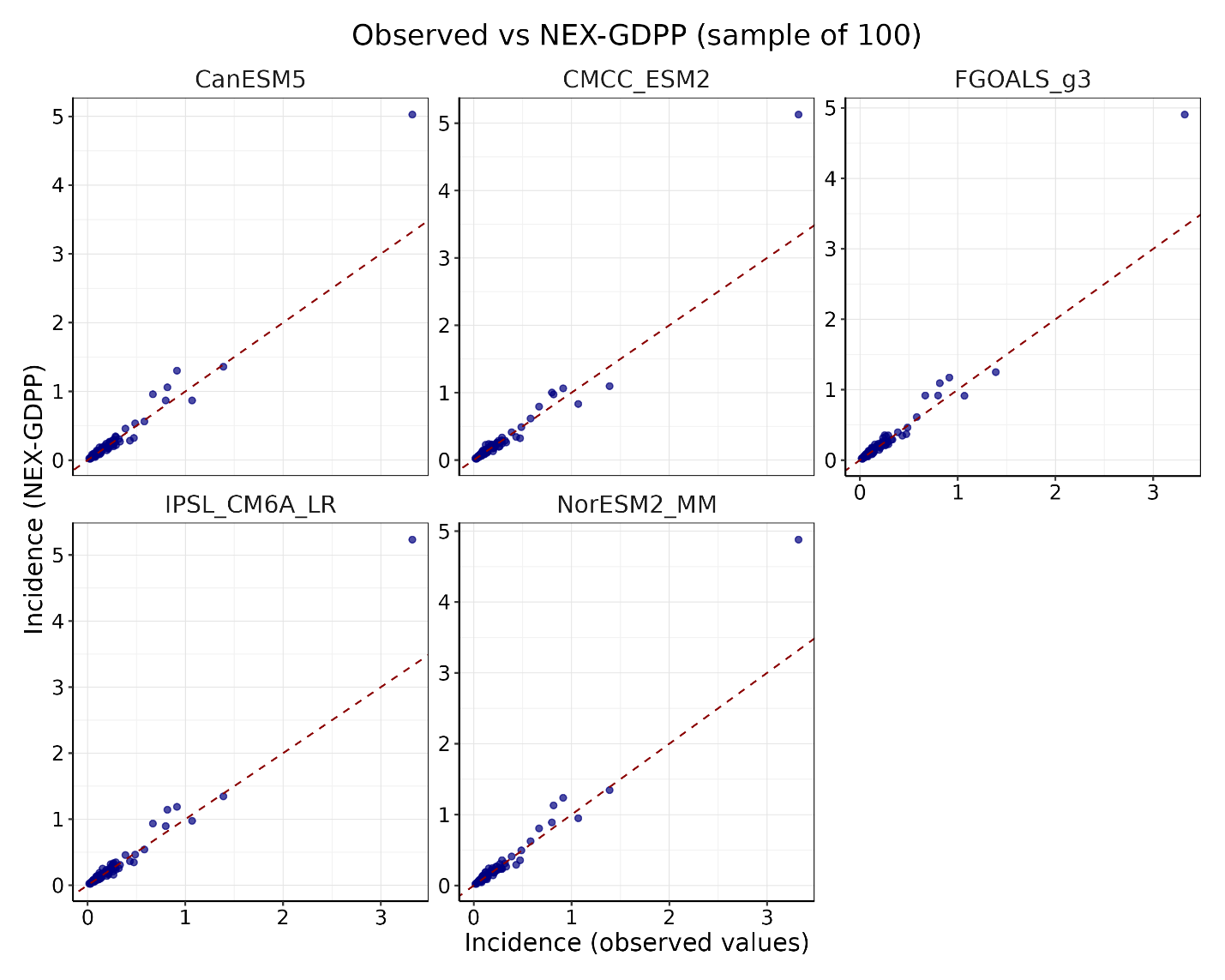


**Figure S16:** **Time series of predicted leptospirosis incidence from all models for four randomly selected NUTS 3 regions (reference period, 2010-2023)**

**
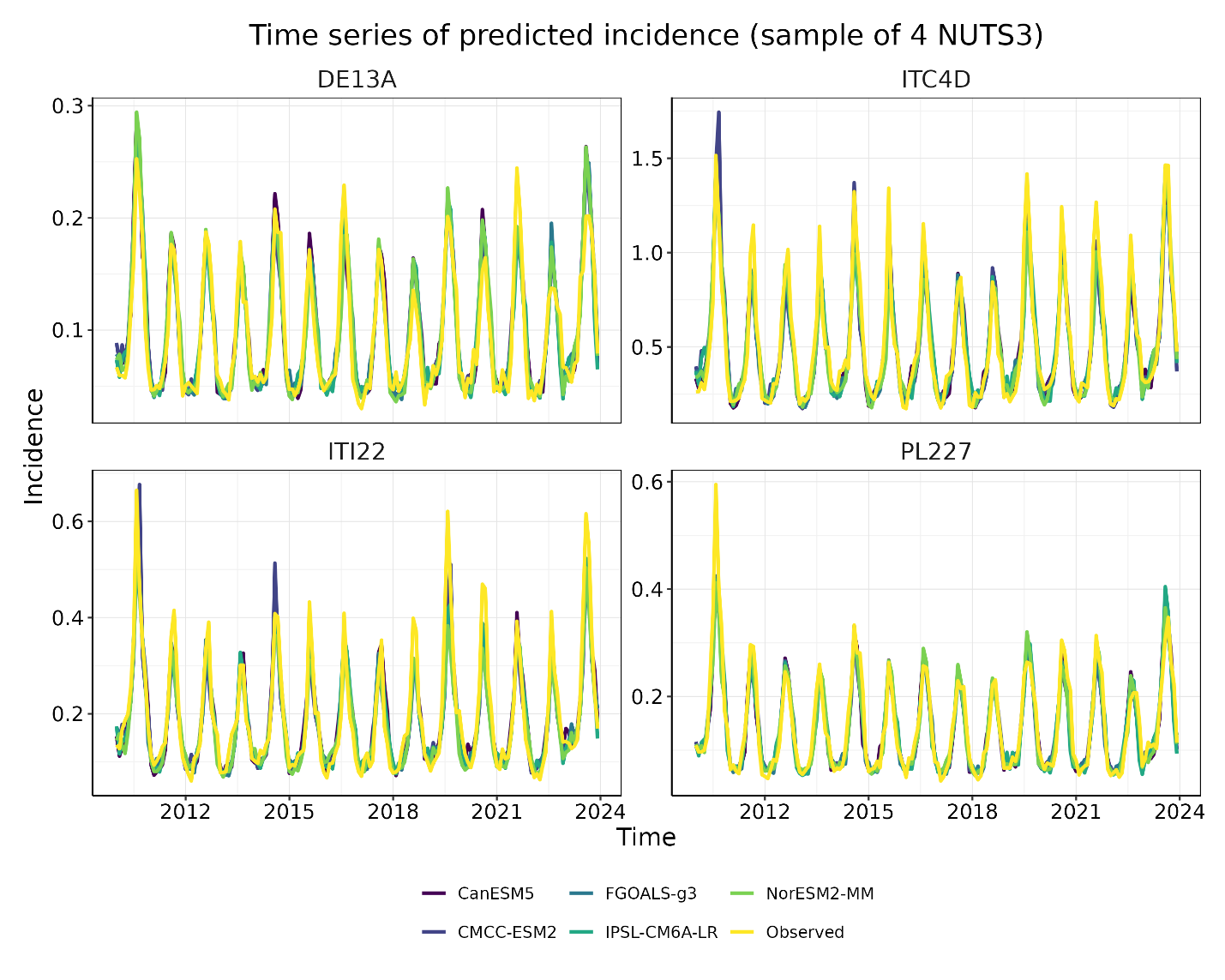
**

We calculated the average monthly incidence across all months and years, based on the ensemble mean of all NEX-GDDP models (Figure S17-S20).

**Figure S17:** **Spatial distribution of leptospirosis incidence rates (cases per 100,000 population) across Europe at the NUTS 3 level, based on monthly averages under** **SSP2-4.5 (short-term future, 2041-2060)**

**
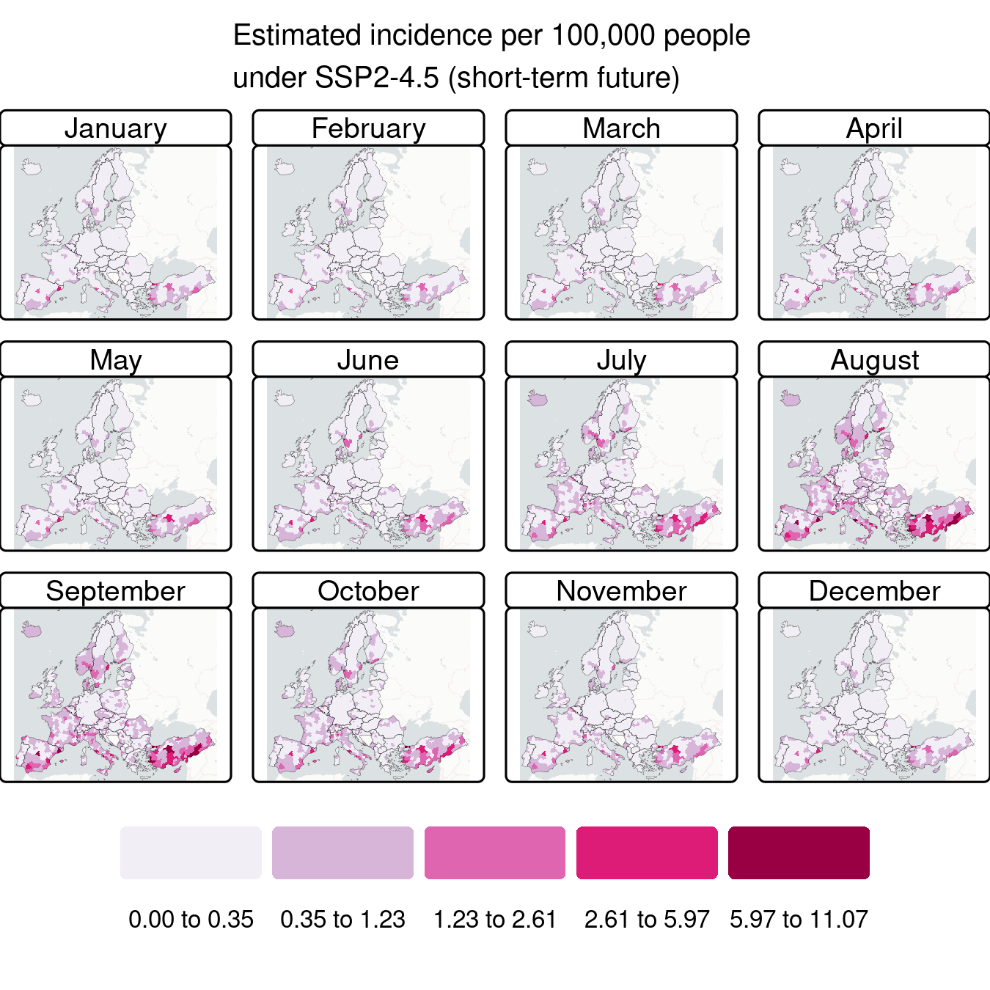
**

**Figure S18:** **Spatial distribution of leptospirosis incidence rates (cases per 100,000 population) across Europe at the NUTS 3 level, based on monthly averages under** **SSP2-4.5 (long-term future, 2081-2100)**

**
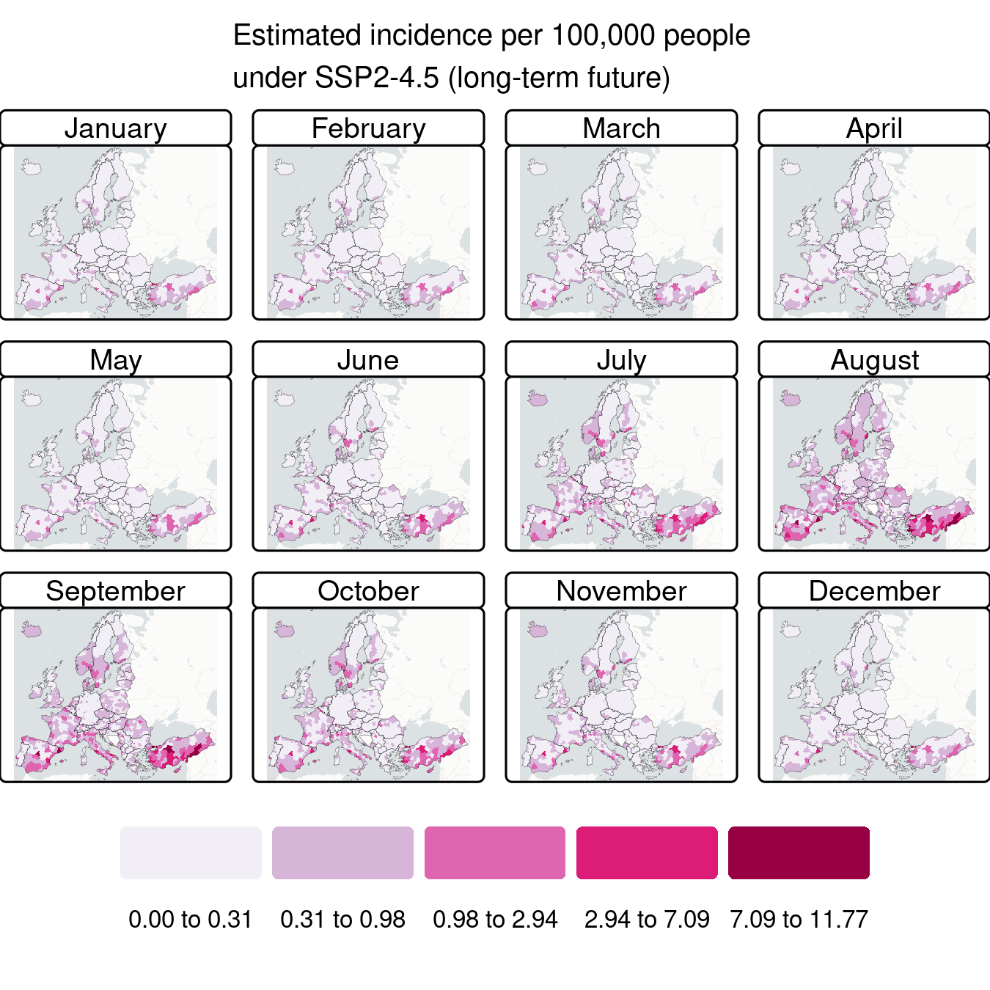
**

**Figure S19:** **Spatial distribution of leptospirosis incidence rates (cases per 100,000 population) across Europe at the NUTS 3 level, based on monthly averages under** **SSP5-8.5 (short-term future, 2041-2060)**

**
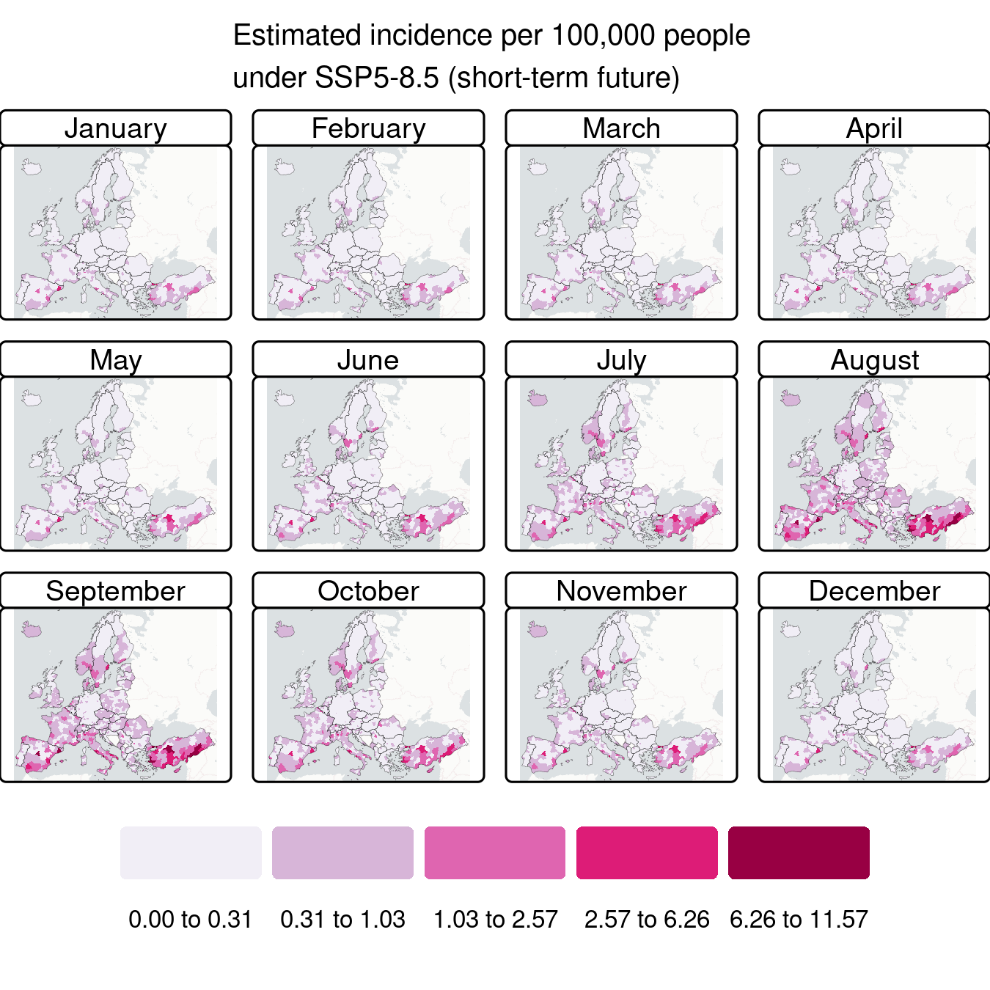
**

**Figure S20:** **Spatial distribution of leptospirosis incidence rates (cases per 100,000 population) across Europe at the NUTS 3 level, based on monthly averages under** **SSP5-8.5 (long-term future, 2081-2100)**


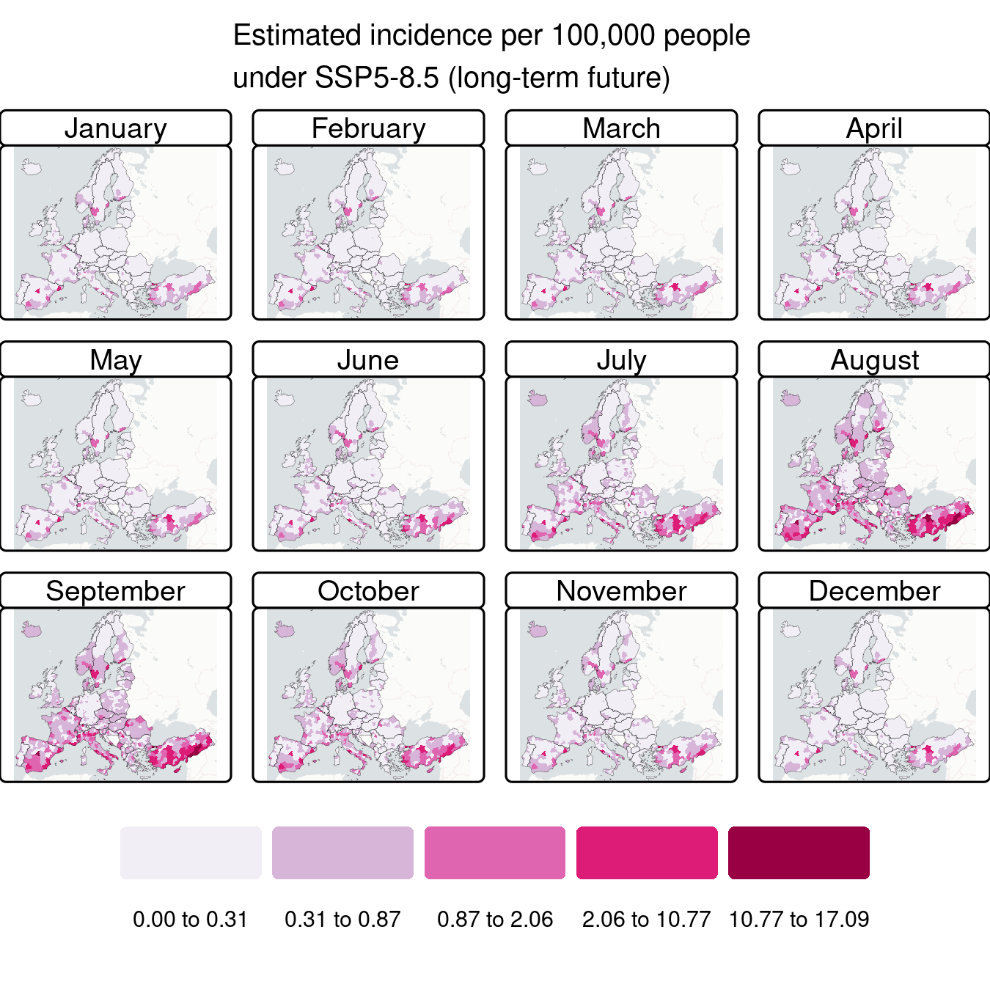


To characterise uncertainty, we also estimated the monthly SD and the CV (Figure S21-S28).

**Figure S21: Model uncertainty across NUTS 3 regions, expressed as the standard deviation of predicted incidence under SSP2-4.5 (short-term future, 2041-2060)**

**
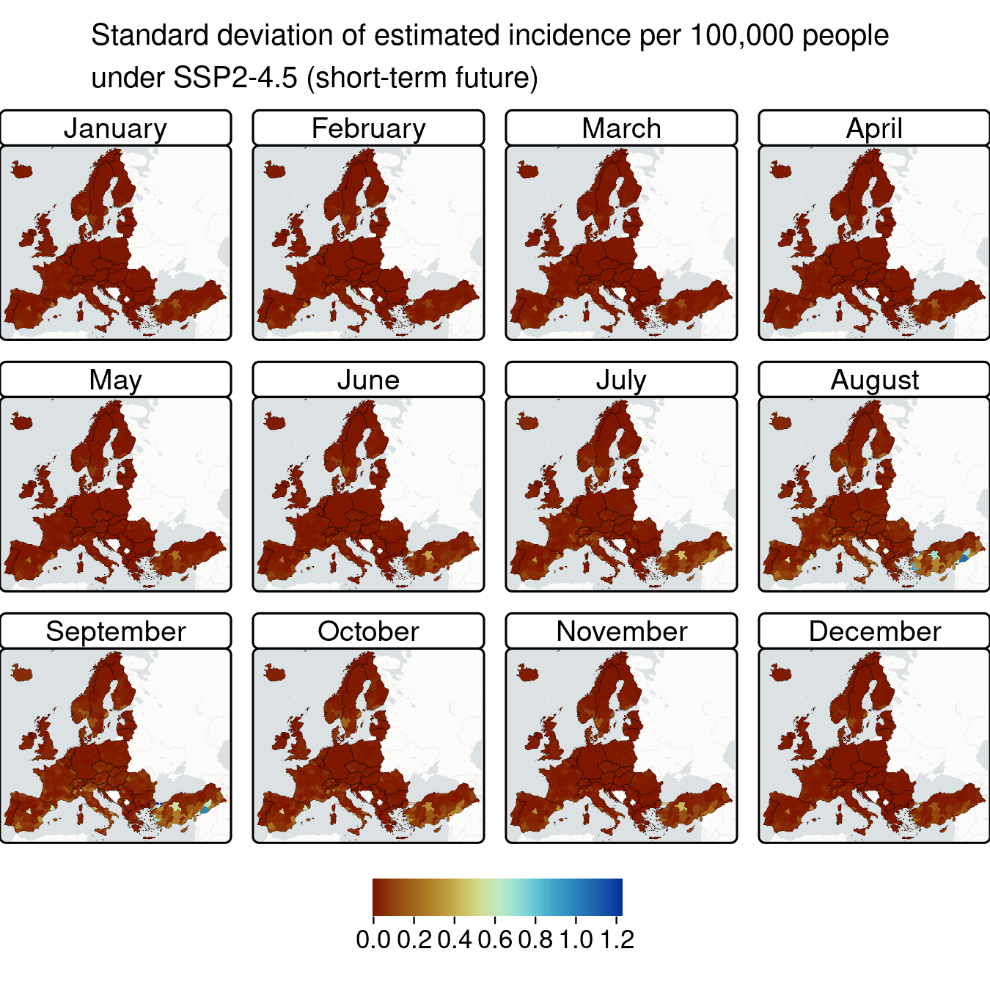
**

**Figure S22: Model uncertainty across NUTS 3 regions, expressed as the coefficient of variation of predicted incidence under SSP2-4.5 (short-term future, 2041-2060)**

**
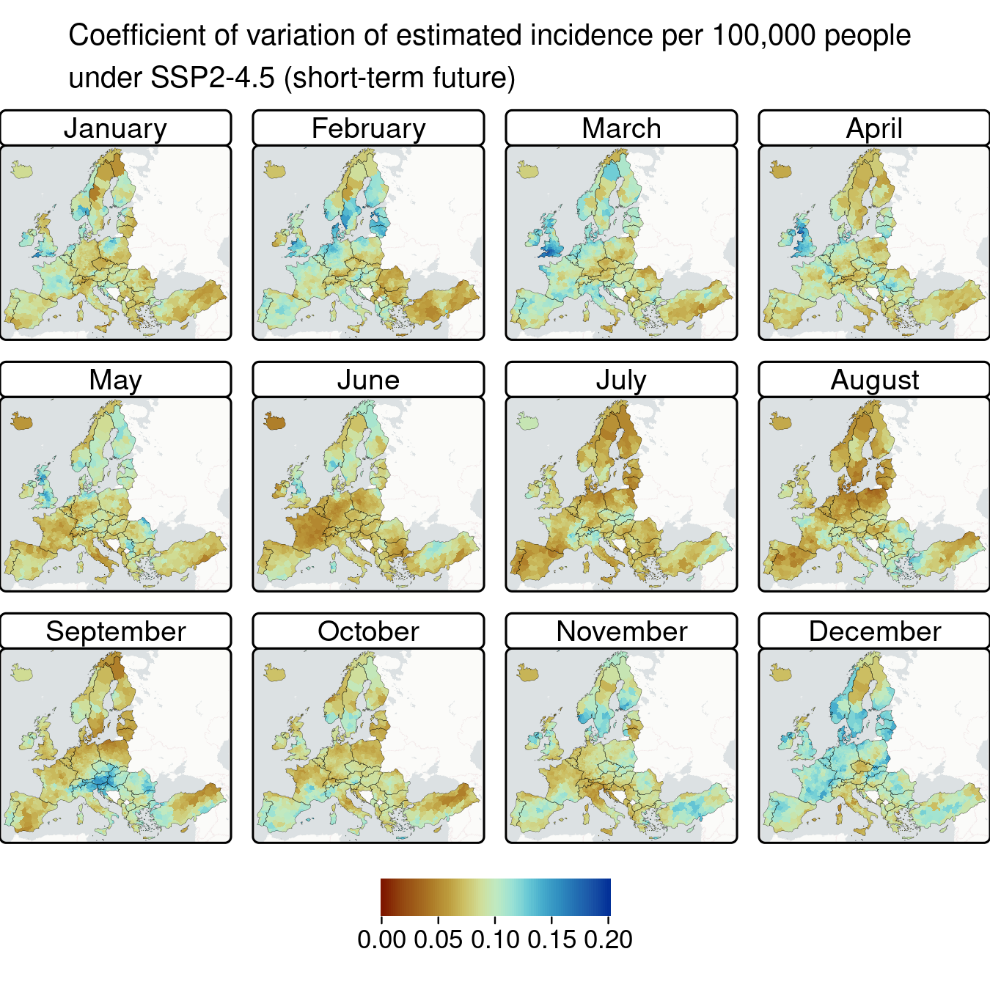
**

**Figure S23: Model uncertainty across NUTS 3 regions, expressed as the standard deviation of predicted incidence under SSP2-4.5 (long-term future, 2081-2100)**

**
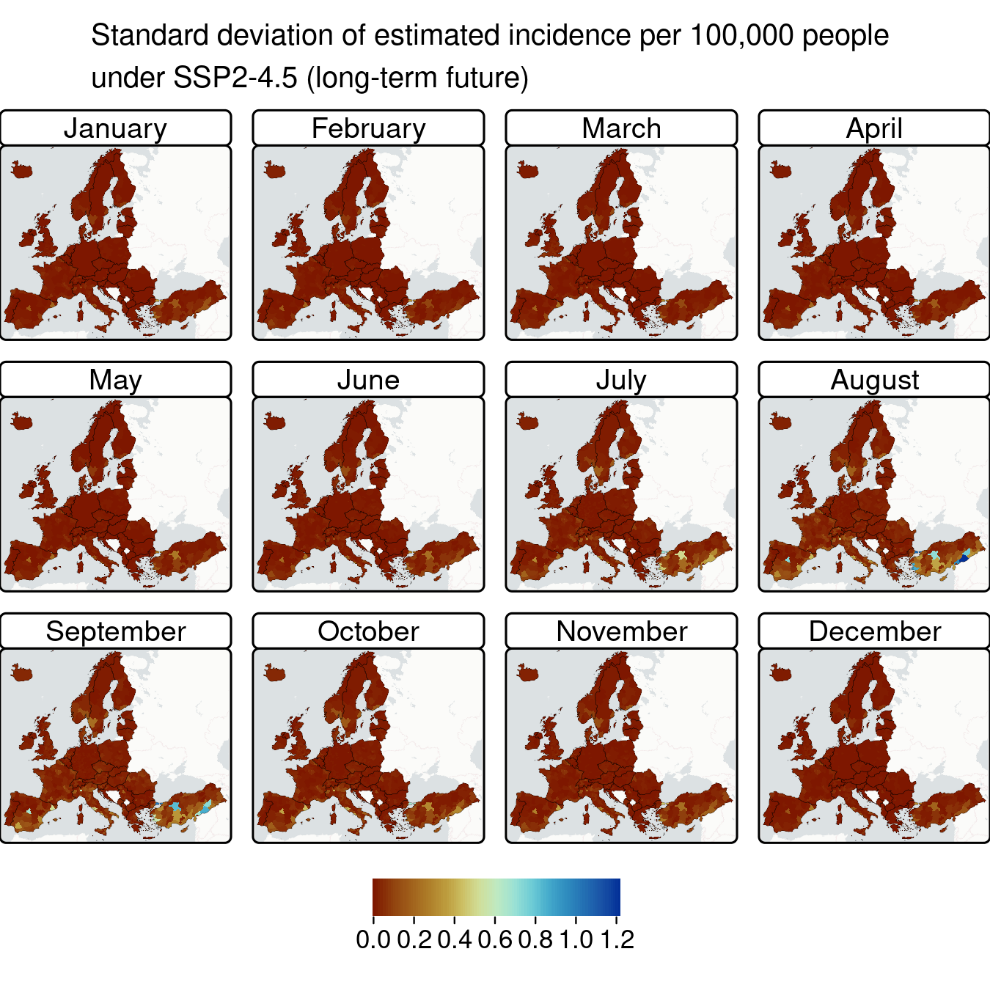
**

**Figure S24: Model uncertainty across NUTS 3 regions, expressed as the coefficient of variation of predicted incidence under SSP2-4.5 (long-term future, 2081-2100)**

**
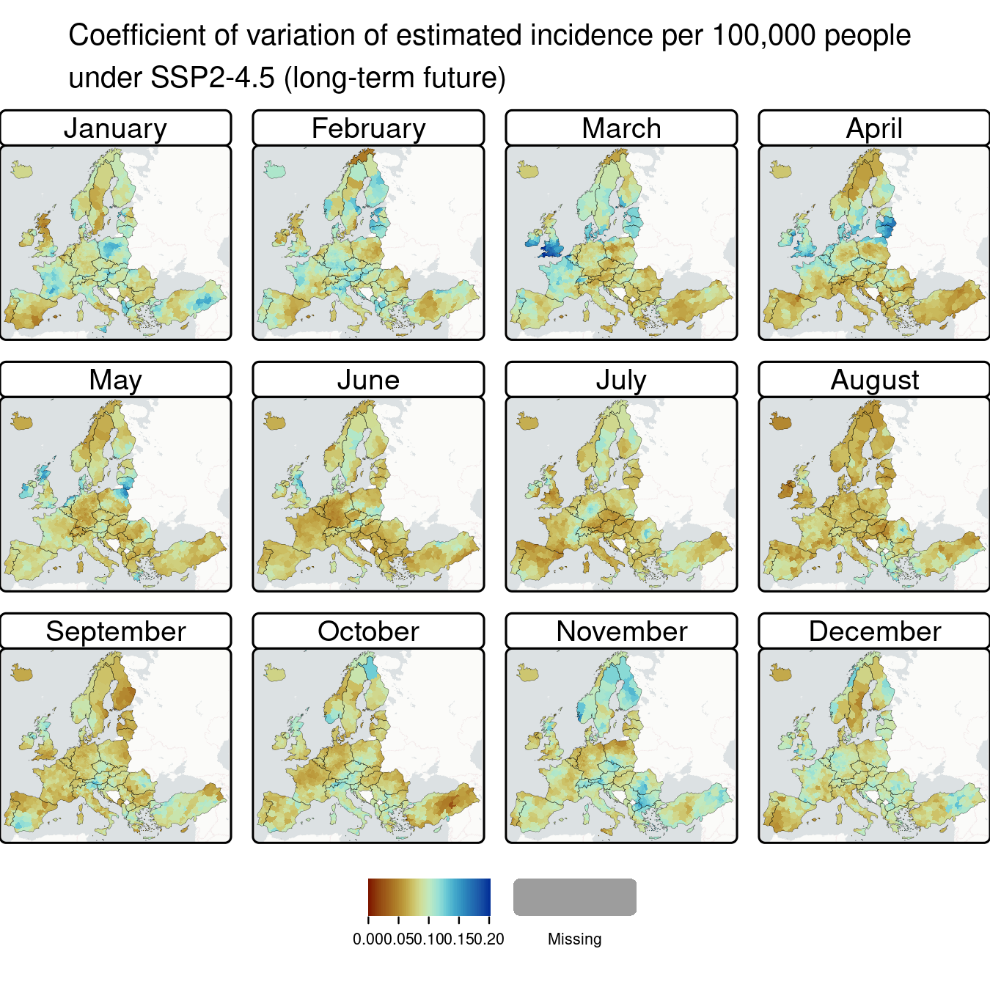
**

**Figure S25: Model uncertainty across NUTS 3 regions, expressed as the standard deviation of predicted incidence under SSP5-8.5 (short-term future, 2041-2060)**

**
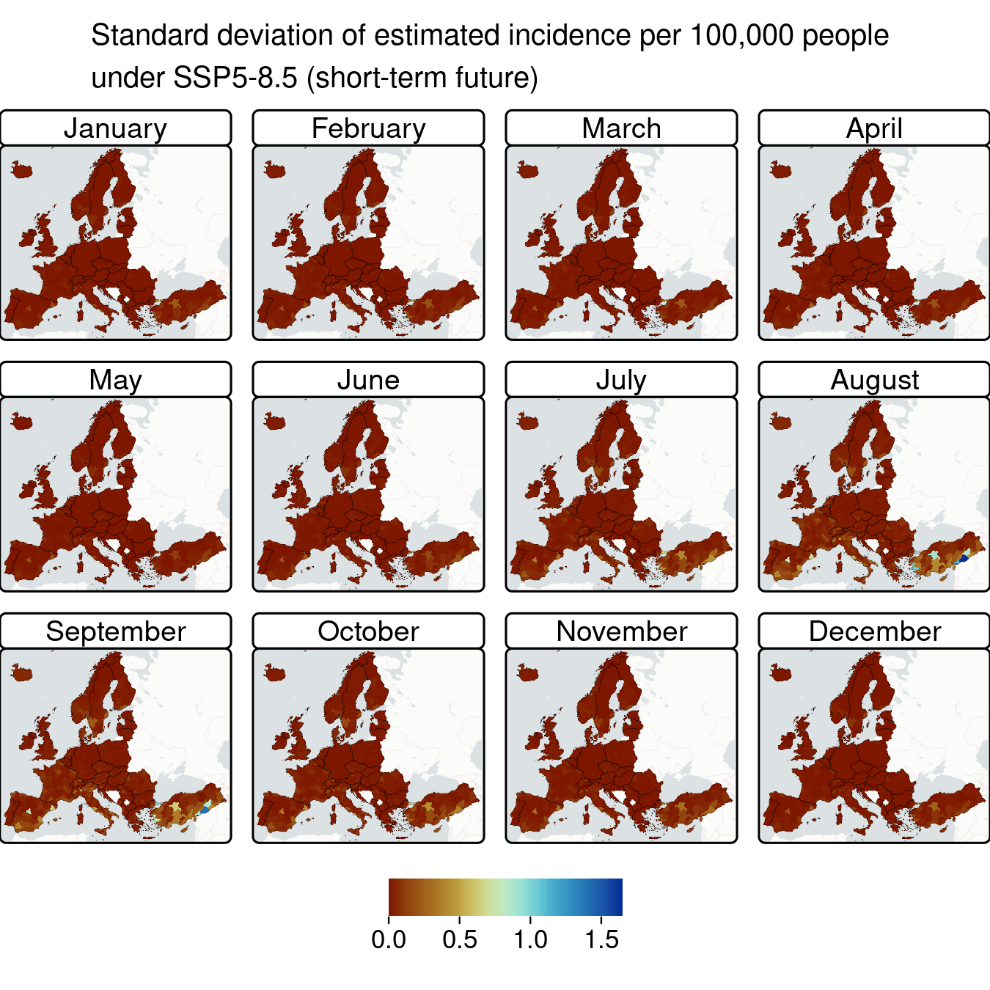
**

**Figure S26: Model uncertainty across NUTS 3 regions, expressed as the coefficient of variation of predicted incidence under SSP5-8.5 (short-term future, 2041-2060)**

**
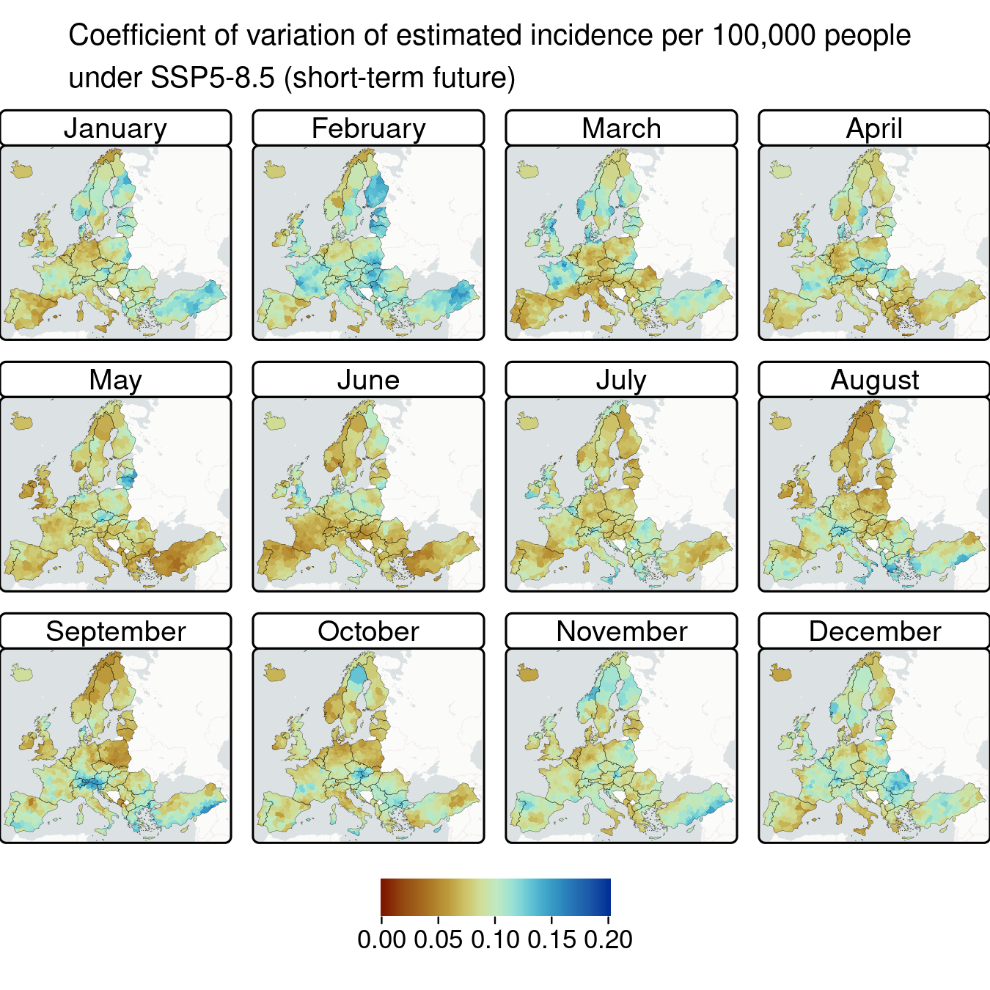
**

**Figure S27: Model uncertainty across NUTS 3 regions, expressed as the standard deviation of predicted incidence under SSP5-8.5 (long-term future, 2081-2100)**

**
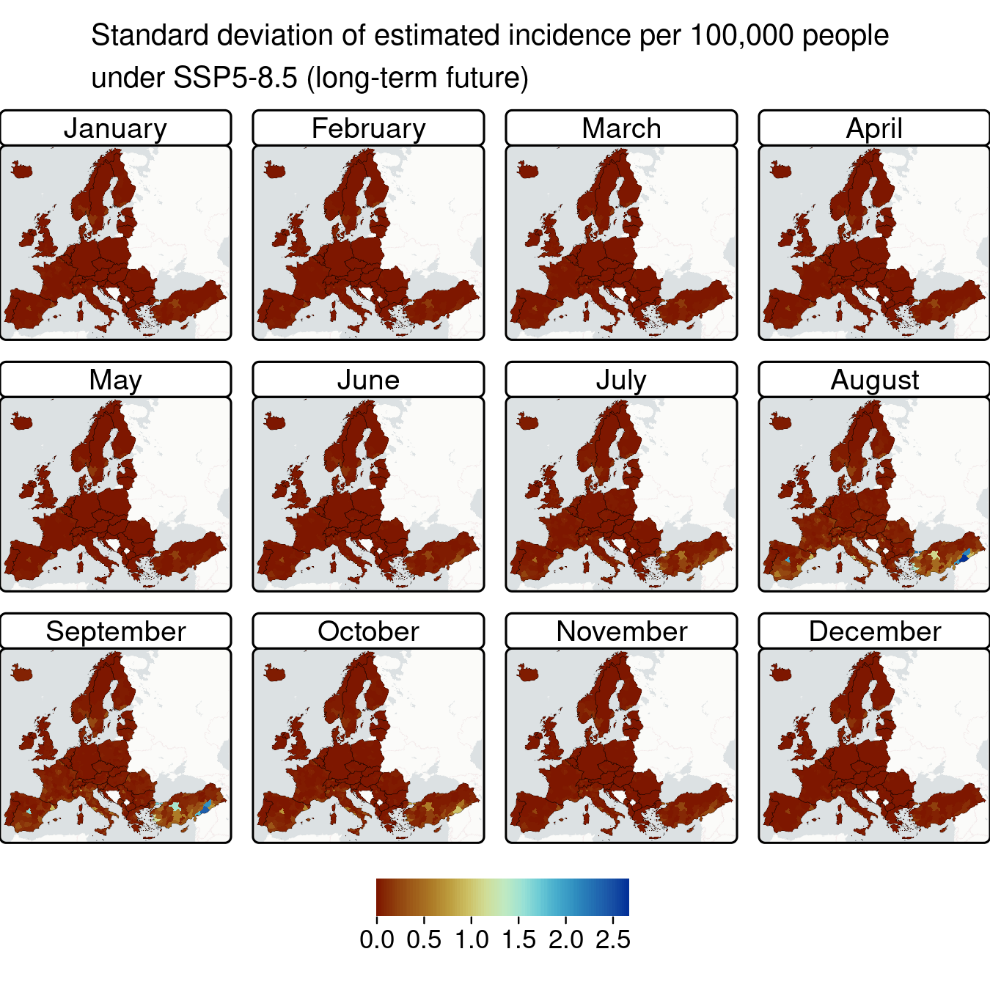
**

**Figure S28: Model uncertainty across NUTS 3 regions, expressed as the coefficient of variation of predicted incidence under SSP5-8.5 (long-term future,2081-2100)**

**
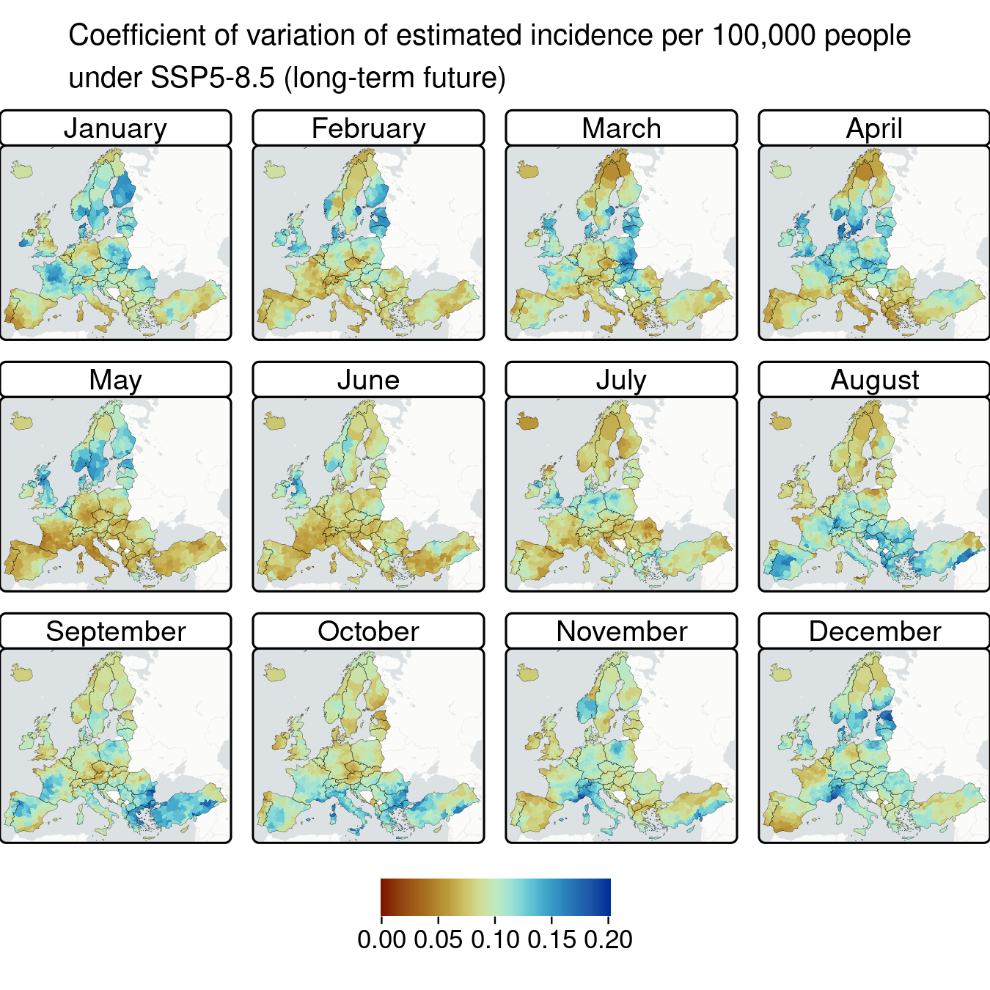
**

Estimates under future scenarios for four randomly chosen NUTS 3 are shown in Figure S29-S42.

**Figure S29:** **Monthly incidence (purple) and 95% confidence intervals (lavender) for four randomly selected NUTS 3 regions.** Data are based on the ensemble of all NEX-GDDP models over SSP2-4.5 (short-term future, 2041-2060)


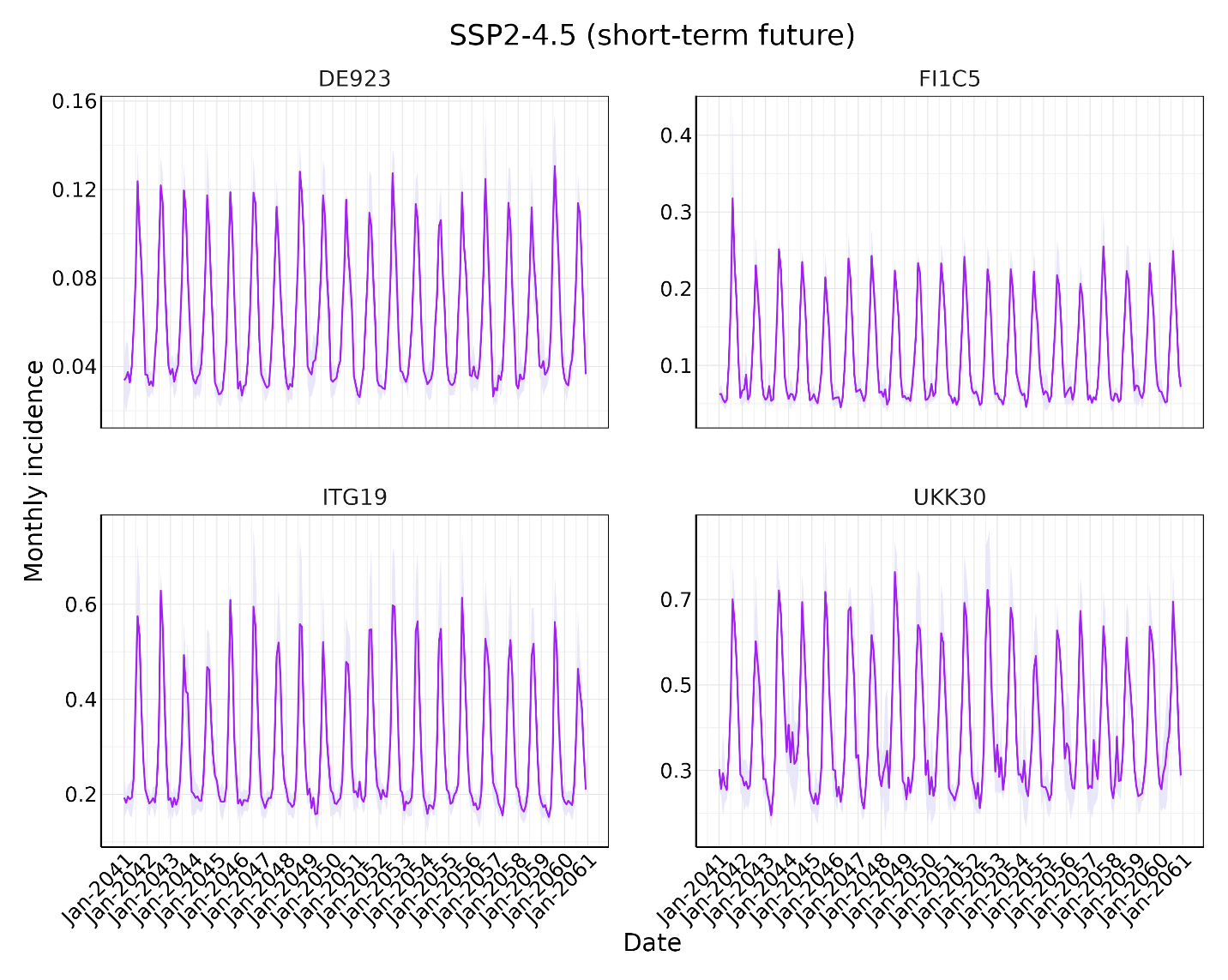


**Figure S40:** **Monthly incidence (purple) and 95% confidence intervals (lavender) for four randomly selected NUTS 3 regions.** Estimates represent the ensemble mean, calculated across all NEX-GDDP models over SSP2-4.5 (long-term future, 2081-2100)
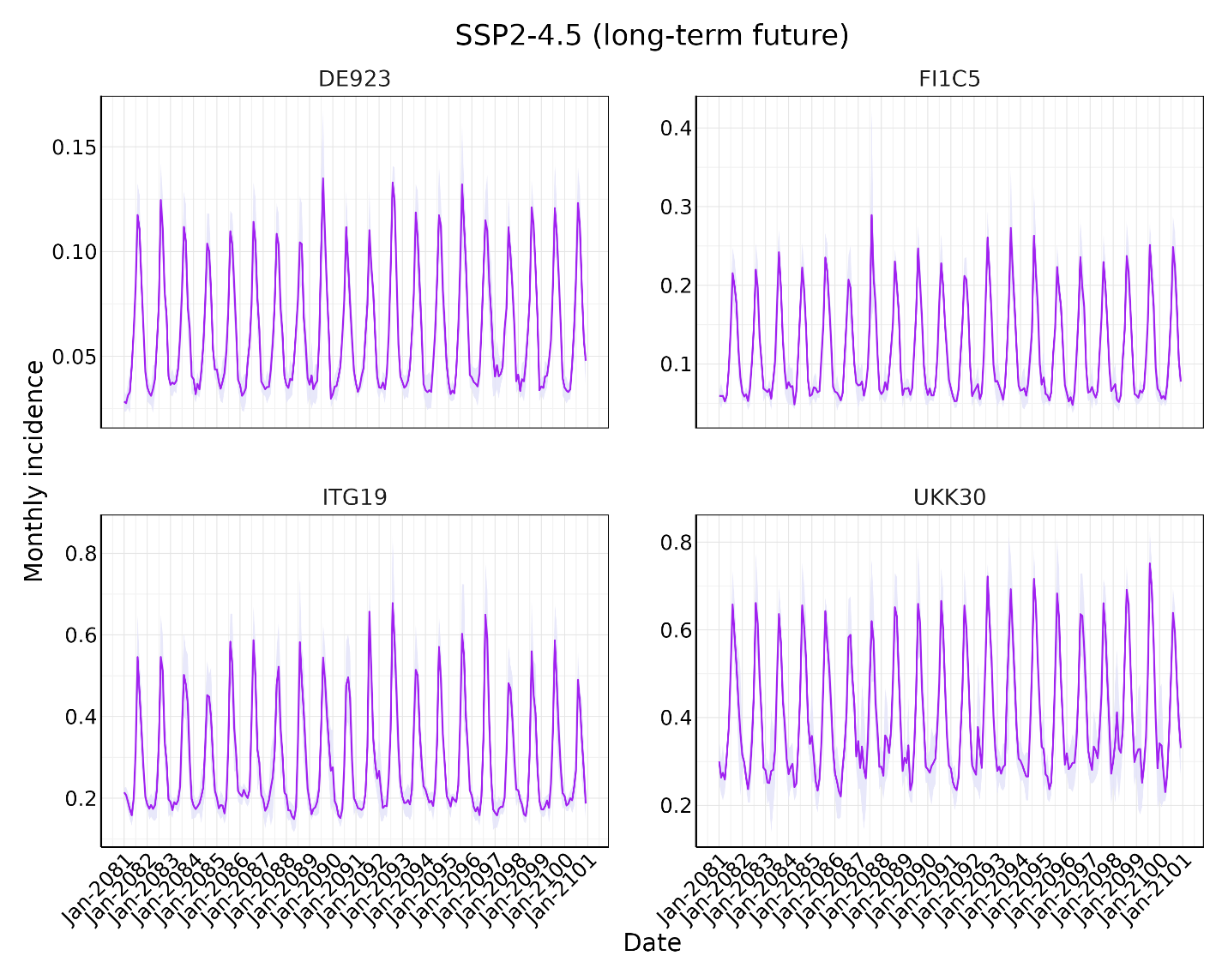


**Figure S41:** **Monthly incidence (purple) and 95% confidence intervals (lavender) for four randomly selected NUTS 3 regions.** Estimates represent the ensemble mean, calculated across all NEX-GDDP models over SSP5-8.5 (short-term future, 2041-2060)


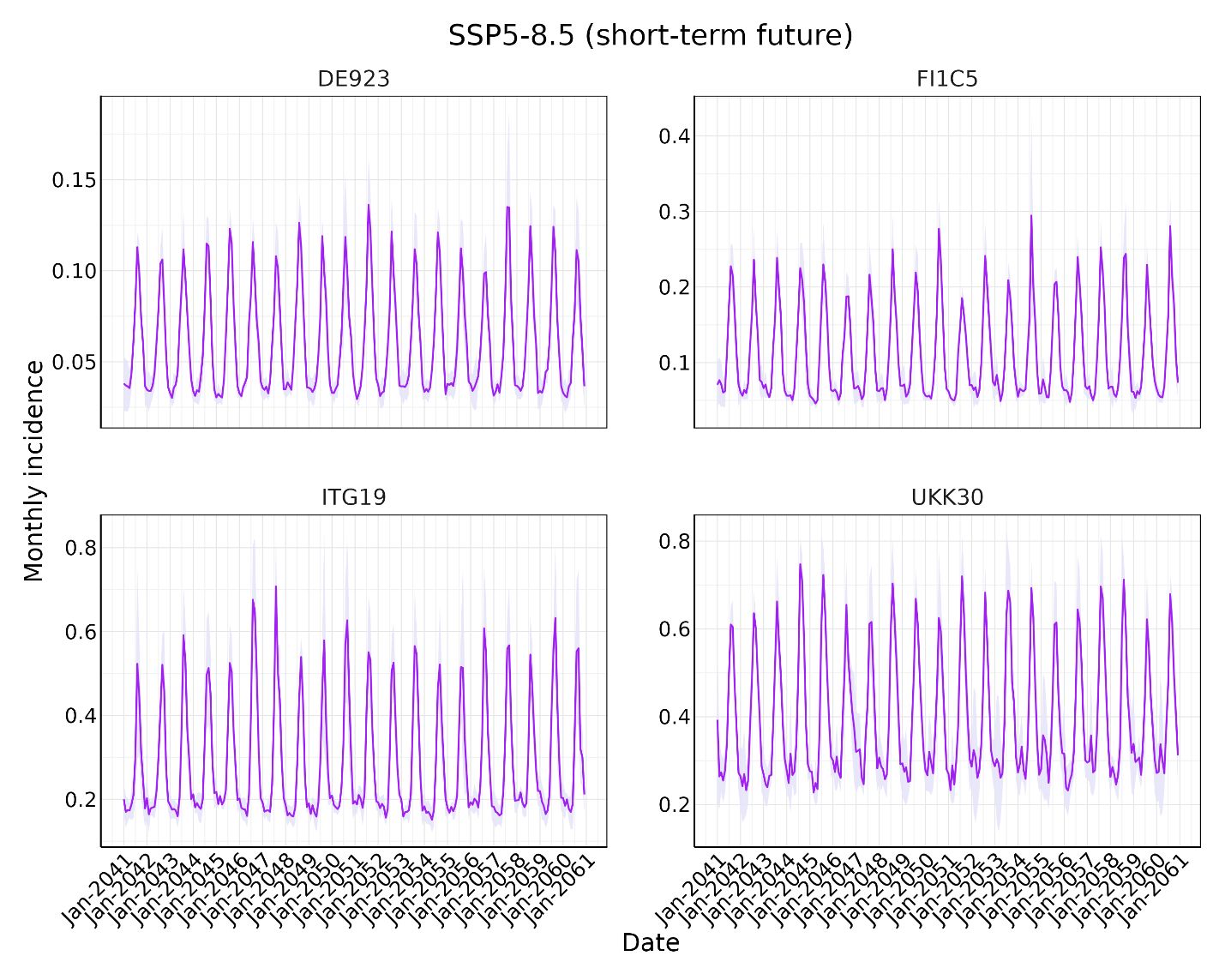


**Figure S42:** **Monthly incidence (purple) and 95% confidence intervals (lavender) for four randomly selected NUTS 3 regions.** Estimates represent the ensemble mean, calculated across all NEX-GDDP models over SSP5-8.5 (long-term future,2081-2100)


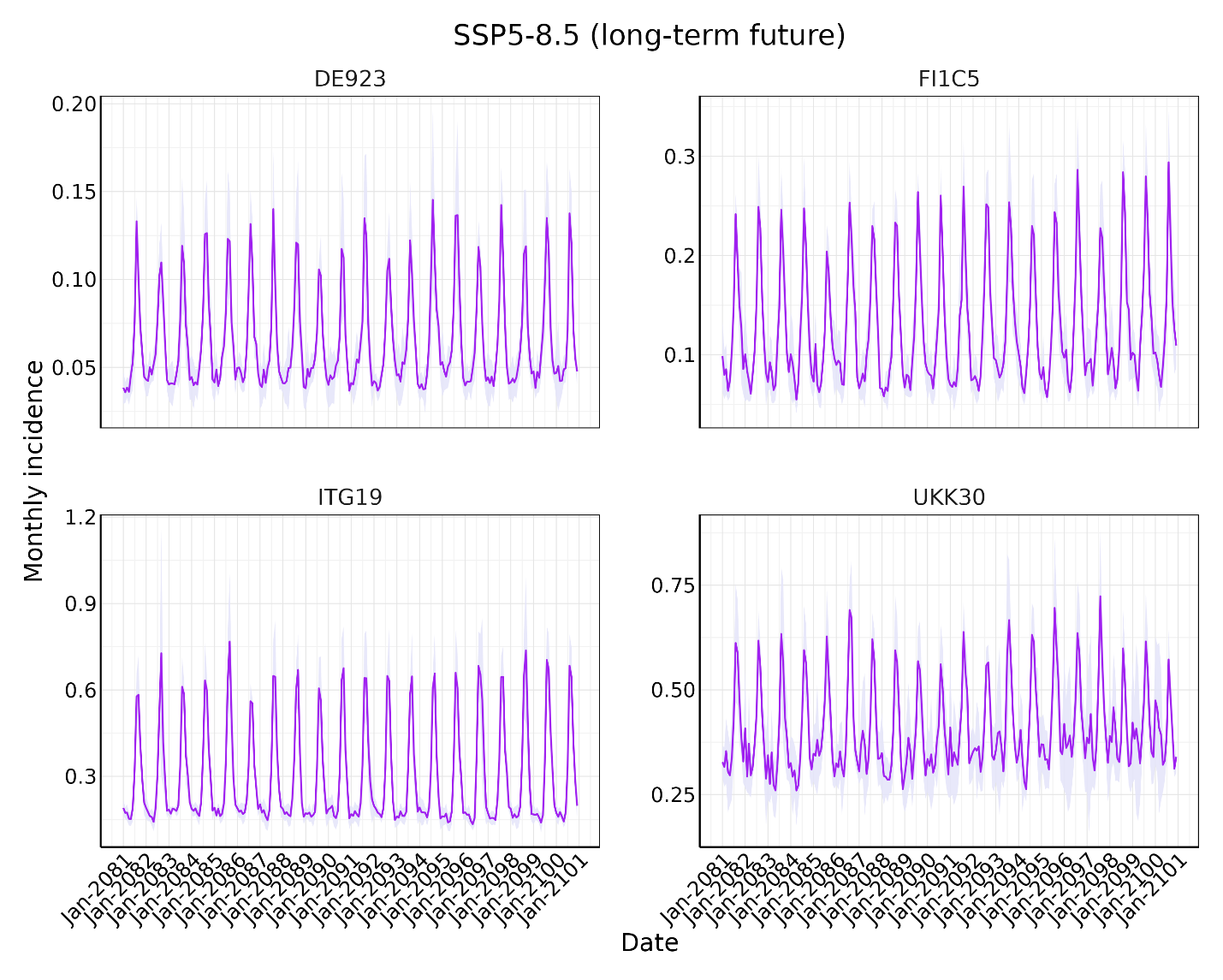


In Figure S43 and S44 we present the estimated incidence for highly populated NUTS 3 regions for SSP2-4.5 and SSP5-8.5 respectively. The estimated incidence is heterogeneous across the NUTS and peaks in August–September, with some months showing decreases in the future scenarios compared with the historical-reference period. Overall, these results highlight strong regional and seasonal variability and underscore the need for tailored, location-specific planning and response strategies.

**Figure S43: Estimated monthly leptospirosis incidence for highly populated NUTS 3 regions under SSP2-4.5**

**
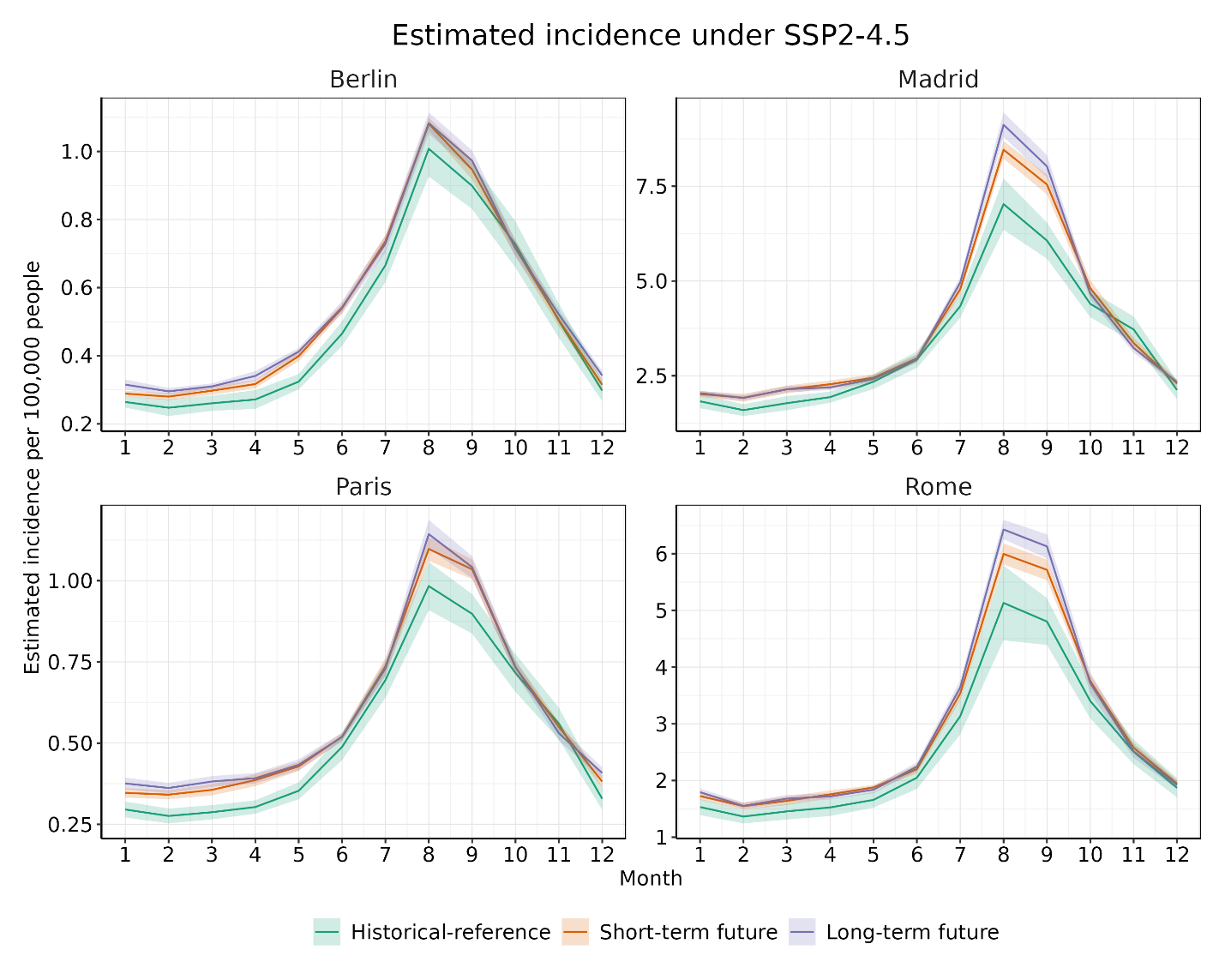
**

**Figure S44: Estimated monthly leptospirosis incidence for highly populated NUTS 3 regions under SSP5-8.5**

**
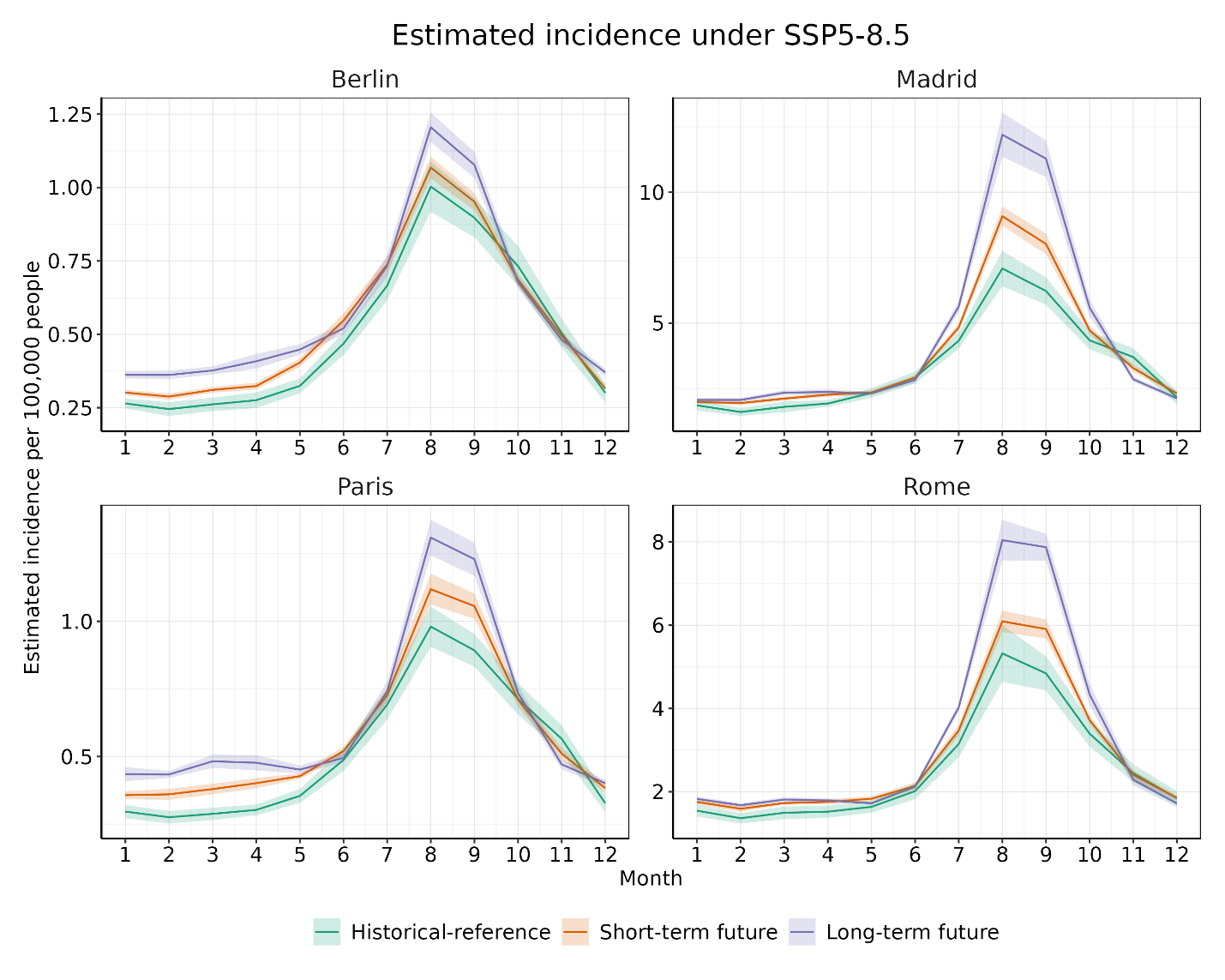
**
